# Solvent Suppression in Pure Shift NMR

**DOI:** 10.1021/acs.analchem.3c05379

**Published:** 2024-02-21

**Authors:** Emma L. Gates, Jonathan P. Bradley, Daniel B. G. Berry, Mathias Nilsson, Gareth A. Morris, Ralph W. Adams, Laura Castañar

**Affiliations:** † Department of Chemistry, University of Manchester, Oxford Road, Manchester M13 9PL, U.K.; ‡ Johnson Matthey Technology Centre, Blounts Court Road, Sonning Common RG4 9NH, U.K.; § Department of Organic Chemistry, Faculty of Chemical Science, Complutense University of Madrid, 28040 Madrid, Spain

## Abstract

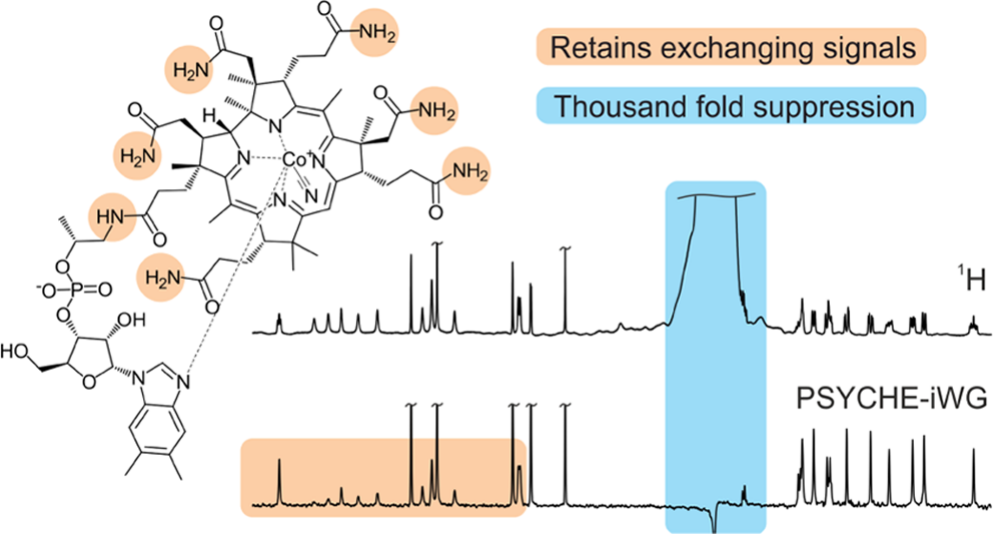

Intense solvent signals in ^1^H solution-state NMR experiments typically cause severe distortion of spectra and mask nearby solute signals. It is often infeasible or undesirable to replace a solvent with its perdeuterated form, for example, when analyzing formulations in situ, when exchangeable protons are present, or for practical reasons. Solvent signal suppression techniques are therefore required. WATERGATE methods are well-known to provide good solvent suppression while enabling retention of signals undergoing chemical exchange with the solvent signal. Spectra of mixtures, such as pharmaceutical formulations, are often complicated by signal overlap, high dynamic range, the narrow spectral width of ^1^H NMR, and signal multiplicity. Here, we show that by combining WATERGATE solvent suppression with pure shift NMR, ultrahigh-resolution ^1^H NMR spectra can be acquired while suppressing intense solvent signals and retaining exchangeable ^1^H signals. The new method is demonstrated in the analysis of cyanocobalamin, a vitamin B12 supplement, and of an eye-drop formulation of atropine.

Stringent regulations apply to the production and marketing of pharmaceuticals to ensure that any adverse effects from their use are identified and minimized. Typically, full characterization of all active pharmaceutical ingredients (APIs), excipients, and impurities is required for components in excess of 0.1%.^1^^1^H solution-state NMR spectroscopy is widely used for characterization and quantification purposes in such cases. Chemical shift information and scalar coupling constants are frequently used in NMR spectroscopy to aid in molecular structure elucidation. However, for complex systems such as pharmaceutical formulations, ^1^H NMR spectra are often complicated by low chemical shift dispersion, signal multiplicity patterns, and the concentration disparity between major compounds and impurities, which combine to cause significant spectral overlap and hinder spectral analysis. ^1^H NMR spectra are particularly complicated by the presence of intense signals from nondeuterated solvent (such as H_2_O), where the high dynamic range between the intense solvent signal and weak solute signals makes the latter difficult to observe. Pharmaceutical formulations, metabolomic samples, and biological samples are routinely analyzed in nondeuterated solvents either to mimic in vivo conditions, to retain signals from exchangeable protons, or to avoid perturbing the system of interest (for example, to avoid denaturing proteins).^2^

^1^H NMR spectra can be simplified by acquiring spectra containing only chemical shift information, using “pure shift” experiments.^3−6^ These increase spectral resolution by collapsing multiplet structures into a single line at the chemical shift of each proton environment. There are multiple types of 1D ^1^H pure shift experiment, which differ in the treatment of active (observed) and passive (coupled) spins. A *J*-refocusing element, composed of a hard 180° radiofrequency (RF) pulse and an active spin-refocusing (ASR) element, when placed in the middle of an evolution time, refocuses the scalar coupling evolution of active spins while allowing the chemical shift to evolve. High-sensitivity pure shift spectra can be obtained using a homonuclear band-selective decoupling (BS)^7,8^ ASR element. Broadband homonuclear decoupled spectra can be acquired using the Zangger–Sterk (ZS),^9^ bilinear rotation decoupling (BIRD),^10^ or pure shift yielded by chirp excitation (PSYCHE)^11,12^ ASR element, but all three have an intrinsic sensitivity penalty (of 1–2 orders of magnitude depending on the ASR element).^6^

For samples prepared in nondeuterated solvents, additional manipulation of the nuclear magnetization is necessary to suppress the large solvent signal. For samples with large solvent signal intensities, radiation damping^13−16^ becomes a significant problem and can cause intense artifacts throughout the spectrum acquired if adequate solvent suppression is not performed. A common solution to this problem is to use presaturation, to saturate and remove the large solvent signal(s).^17^ Although presaturation methods achieve good solvent suppression, signals from nuclei in chemical exchange with the solvent are also saturated and so are strongly attenuated. This unwanted signal suppression is also observed with the NOESY-presaturation method,^18^ which has recently been combined with pure shift techniques.^19^ Furthermore, presaturation requires a long, low-power continuous wave RF pulse applied during the recovery delay of the NMR experiment in order to saturate the solvent resonance, which can lead to an undesirably long experiment time.

Alternative solvent suppression methods such as excitation sculpting^20^ and WATERGATE (water suppression by gradient-tailored excitation, WG)^21,22^ retain signals from exchangeable nuclei and do not require lengthy recovery delays. Instead, these methods use either selective pulses or binomial series of pulses, in combination with pulsed field gradients (PFGs), to refocus the off-resonance signal while dephasing the on-resonance solute signal. Excitation sculpting has previously been integrated into the PSYCHE experiment;^11^ however, that implementation provides poor solvent suppression. A WATERGATE pure shift method has also been reported in which WATERGATE was concatenated with the PSYCHE triple spin echo (TSE) experiment (W5_*n*_-TSE-PSYCHE where *n* represents the number of W5 elements).^23^ Although good solvent suppression has been reported with this method, the addition of separate WATERGATE elements causes unwanted scalar coupling evolution and leads to greatly increased chunking sidebands (Supporting Information, Section E).

Here, an integrated WATERGATE (iWG) pure shift experiment is introduced, and its performance is compared to that of previously published methods. The proposed pure shift iWG experiment enables retention of exchangeable proton signals, allows the use of short recovery delays for either fast data acquisition or acquisition of higher signal-to-noise ratio (SNR) spectra per unit time, and offers over a 1000-fold reduction in solvent signal intensity. As the WATERGATE element is integrated into the pure shift experiment, no extra scalar coupling evolution occurs. The usefulness of the proposed method is demonstrated in the analysis of cyanocobalamin (manufactured vitamin B12), where WATERGATE enables exchangeable amide signals to be observed in the pure shift spectra, and atropine eye-drop solution (a pharmaceutical formulation), where the ultrahigh resolution afforded by the new solvent-suppressed pure shift method aids the identification and characterization of degradation impurities in the atropine formulation.

## Experimental Section

### Sample Preparation

Cyanocobalamin (5 mM) was dissolved in 630 μL of phosphate buffer (H_2_O, pH 8.1, 0.1 M) with 70 μL of D_2_O added for locking purposes. 630 μL of a commercial sample of 1% atropine sulfate ophthalmic solution was added to 70 μL of D_2_O.

### NMR Experiments

Spectra were recorded on a Bruker Avance 500 NEO spectrometer with a 5 mm room temperature triaxial gradient TBI probe with a maximum nominal *G*_Z_ gradient strength of 67 G cm^–1^, and the results were processed using the Bruker Topspin software package (version 4.1.4).

All pure shift spectra shown in the main paper and Supporting Information were acquired with the following parameters unless otherwise stated. Sixteen increments of 1024 complex data points each were acquired with a spectral width of 10 kHz (20 ppm) and a recovery delay of 3 s, and the first 200 complex data points of each free induction decay were taken to construct the pure shift interferogram. The PSYCHE ASR element was used, with two saltire pulses of 30 ms duration, 20° flip angle β, 10 kHz bandwidth, and a simultaneous PFG of 1.5 G cm^–1^ nominal amplitude. Interferogram construction used the “pshift” macro (https://nmr.chemistry.manchester.ac.uk).

Experiment-specific WATERGATE, presaturation, and NOESY-presaturation parameters are given in figure captions. Further NMR acquisition and processing parameters are detailed in the Supporting Information. All raw data and the Bruker pulse program code used in this work are freely available at DOI: 10.48420/24600114.

## Results and Discussion

### NMR Method

**Figure 1:**
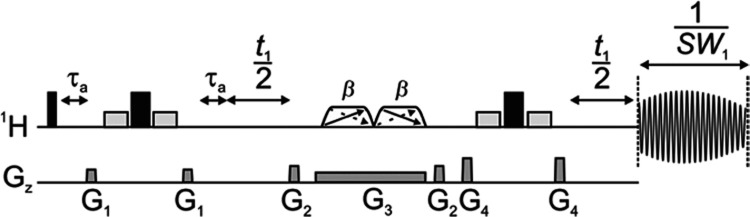
PSYCHE-iWG pulse sequence. Narrow, wide and short rectangles represent hard 90°, hard 180° and selective rectangular 90° radiofrequency pulses, respectively. Trapezoids with two diagonal arrows denote low-power saltire pulses of on-resonance flip angle β (typically 20°). The evolution time *t*_1_ is incremented in the interferogram acquisition mode. The chunk duration is equal to 1/*SW*_1_. τ_A_ controls the time at which *J* evolution is refocused during the chunk, and is equal to 1/4*SW*_1_. Shapes G_1–4_ represent field gradient pulses applied along *z*; G_1_, G_2_ and G_4_ are used for CTP selection and G_3_ is used for spatial encoding. Further details of the pulse sequence are given in the Supporting Information (Section A).

The new PSYCHE-iWG pulse sequence is shown in Figure 1, with more specific details provided in the Supporting Information (Figure S1). The integration of WATERGATE (WG)^21^ into pure shift pulse sequences enables large solvent signals to be suppressed while solute signals are retained, even if in slow/medium chemical exchange with the solvent. Importantly, the appropriate incorporation of the WATERGATE element into a pure shift sequence enables chemical shift evolution to be retained without any net contribution to homonuclear scalar coupling evolution from these elements. The overall scalar coupling evolution is therefore refocused as usual at the midpoint of the data chunk to be acquired.^3−6^ WATERGATE works by applying a zero net rotation to the on-resonance signal, while off-resonance signals experience a 180° refocusing pulse. The PFGs flanking the WATERGATE element enforce the refocusing coherence transfer pathway (CTP), meaning that the on-resonance signal is dephased. The WATERGATE element achieves the desired effect using two low-power selective 90° pulses on either side of a hard 180° pulse. The selective 90° pulses are on resonance for the solvent signal and leave the solute signals unaffected. The overall sequence uses a triple spin echo (TSE)^24^ structure in which the WATERGATE elements refocus off-resonance signals, ensuring that timings remain balanced. The integration of two WATERGATE elements doubles the dephasing of the solvent signal, increasing the extent of the solvent suppression.

**Figure 2:**
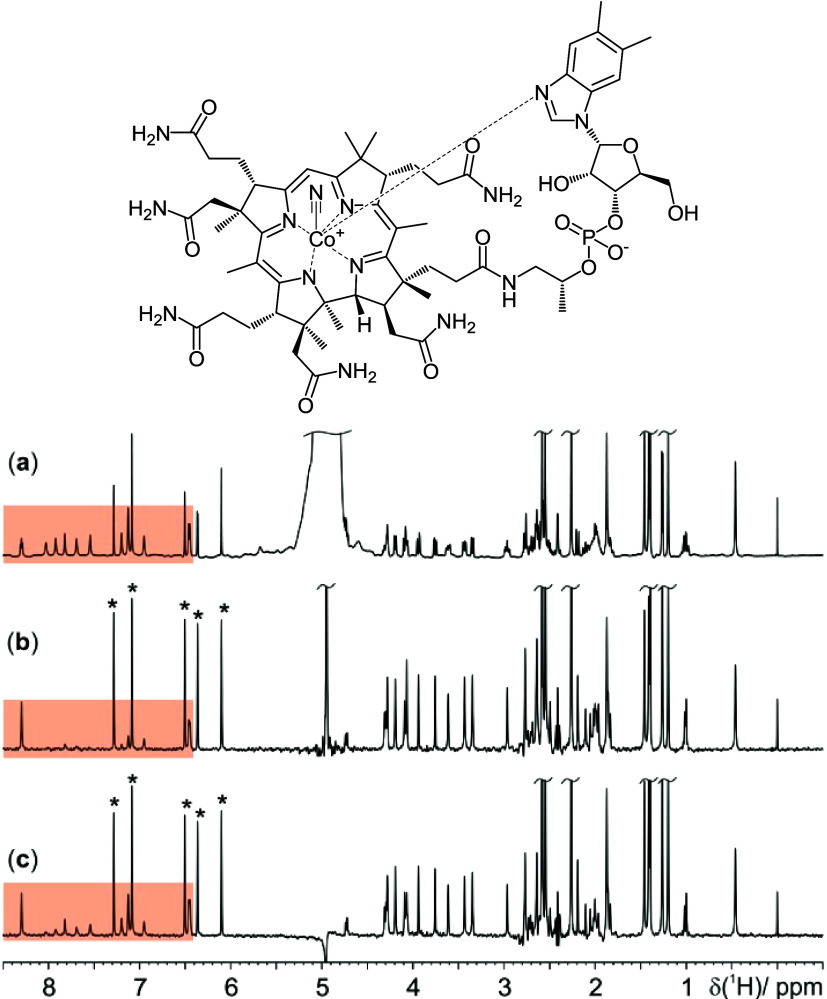
500.13 MHz ^1^H NMR spectra of 5 mM cyanocobalamin in 90:10 H_2_O/D_2_O at 12 °C and pH 8.1. (a) Conventional 1D ^1^H NMR spectrum. (b) NOESY-presaturation PSYCHE spectrum obtained with a presaturation period of 3 s and a NOESY mixing period of 100 ms. (c) PSYCHE-iWG spectrum obtained using 5.5 ms selective rectangular 90° pulses in the WATERGATE element. The PSYCHE element consisted of two saltire pulses of 10 kHz bandwidth, 30 ms duration, and 20° on-resonance flip angle. Sixteen chunks were acquired with a duration of 20 ms. Further experimental details are given in the Supporting Information. The exchangeable protons are highlighted in orange, and the aromatic signals starred.

**Figure 3:**
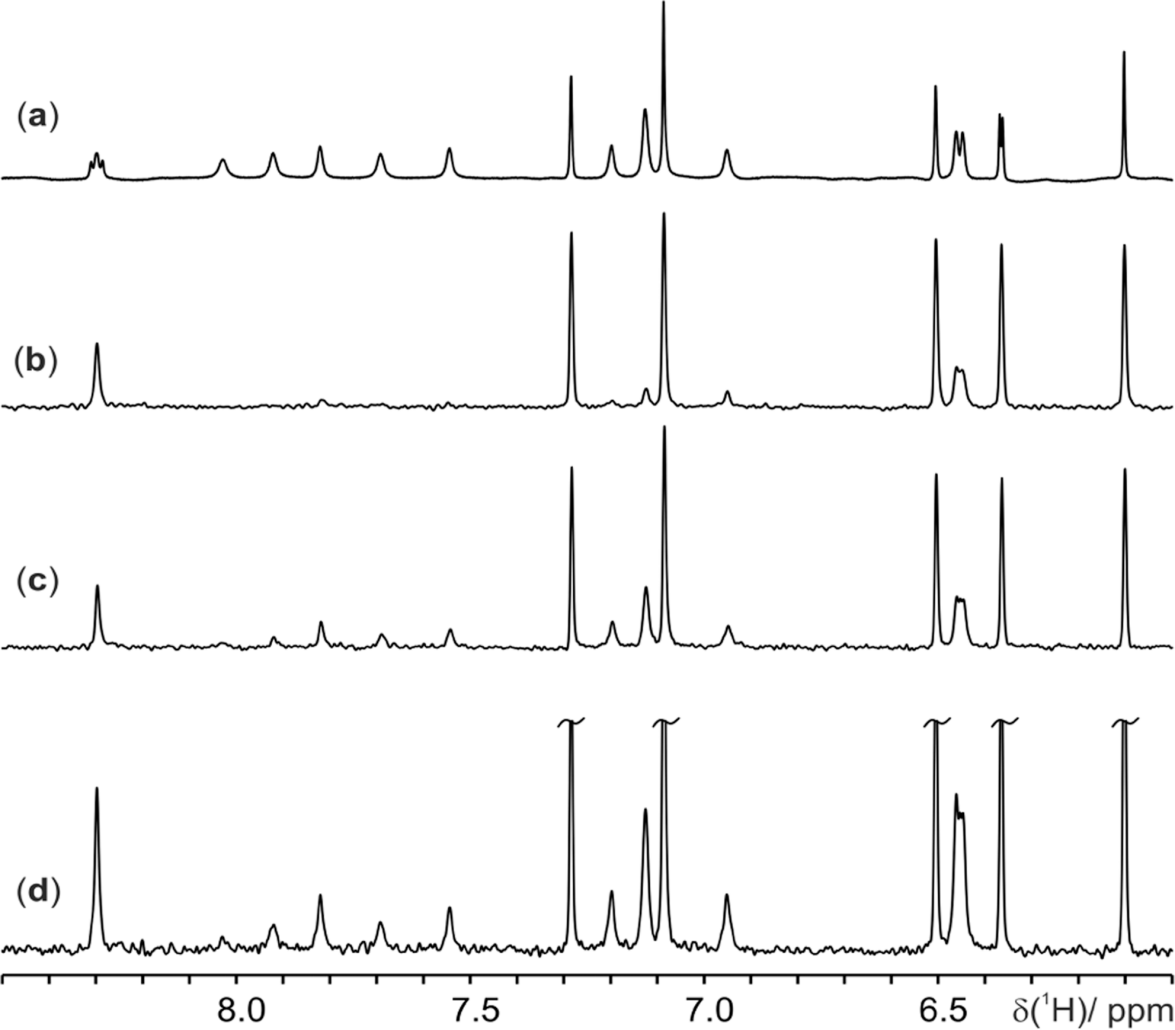
Expansions of 500.13 MHz ^1^H NMR spectra of 5 mM cyanocobalamin in 90:10 H_2_O/D_2_O at 12 °C and pH 8.1. (a) Conventional 1D ^1^H NMR spectrum. (b) NOESY-presaturation PSYCHE spectrum with 32 scans, a presaturation period of 3 s, and a 90° excitation flip angle. (c) PSYCHE-iWG spectrum with 32 scans, a recovery delay of 3 s, and a 90° excitation flip angle. (d) PSYCHE-iWG spectrum with 128 scans, a recovery delay of 0.5 s, and an Ernst angle (73°) excitation pulse. All three PSYCHE experiments had a duration of 30 min. The SNR improvement is detailed in Table S3 in the Supporting Information.

As an alternative to the original WATERGATE element, binominal multiple-pulse WATERGATE elements (e.g., W3/W5)^22^ could be integrated into the sequence. The binominal W5 element comprises a series of varying flip angle pulses with a fixed interpulse delay τ (Supporting Information, Figure S1). It accomplishes the same effect as the WATERGATE element, on-resonance signal experiencing a net 0° rotation. As in the original WATERGATE element, the suppression bandwidth is inversely proportional to the duration. However, the binominal sequence causes suppression not only on resonance but also at intervals of 1/τ Hz. This causes problems if a narrow suppression band is required, because the long interpulse delay τ leads to additional suppression notches within the spectrum of interest, potentially suppressing solute signals (Supporting Information, Figure S2c,d). Although more complicated to set up if the best suppression is required, the selective pulse WATERGATE element is therefore preferable to the binominal W5 element (unless multiple suppression notches are required). The selective pulse WATERGATE element was used in Figures 2 and 3.

Although shown with the PSYCHE ASR in Figure 1, the proposed WATERGATE pure shift experiment is compatible with a range of different ASR elements. The BS element is often used for acquisition of high-sensitivity spectra but is of limited general applicability as it is not broadband. The ZS ASR element enables acquisition of broadband spectra, but typically with lower sensitivity than its PSYCHE counterpart. The BIRD ASR is not appropriate here; it restricts observation to protons directly bonded to ^13^C, so it excludes signals likely to show exchange with solvent and in any case has intrinsic water suppression. The pulse program code (Supporting Information, Section F) enables the user to choose the most appropriate ASR element (BS, ZS, or PSYCHE) for a given sample; see the Supporting Information, Figure S7 for example spectra.

As mentioned earlier, the W5 WATERGATE and PSYCHE pulse sequence elements have been combined previously, by concatenation, for benchtop NMR.^23^ Although such methods can provide excellent solvent suppression, placing the WATERGATE block before the PSYCHE element (Supporting Information, Figure S3d) causes extra, unwanted scalar coupling evolution. This leads to signal discontinuities in the constructed interferogram that Fourier transform to give large undesirable chunking sidebands (Supporting Information, Figure S11b–d). A possible remedy would be to use the perfect-echo version of WATERGATE^25^ in a concatenated WG_*n*_/W5_*n*_-TSE-PSYCHE experiment (Supporting Information, Figures S10 and S11e). This would give a lower SNR than PSYCHE-iWG, but could be preferable in the presence of strong coupling.

### Cyanocobalamin

The corrinoid cyanocobalamin (Figure 2), a synthetic vitamin B12, is used in cases of vitamin B12 deficiency anemia,^26,27^ typically as an oral supplement. Pure shift NMR simplifies the spectrum, collapsing multiplets into singlets and improving resolution, but solvent suppression is required to avoid intense artifacts obscuring the pure shift spectrum. Figure 2 compares spectra acquired using a ^1^H pulse-acquire experiment (Figure 2a), NOESY-presaturation PSYCHE^19^ (Figure 2b), and the new PSYCHE-iWG (Figure 2c).

Both PSYCHE-iWG and NOESY-presaturation PSYCHE offer good solvent signal suppression (2000- and 500-fold, respectively). Eliminating signal overlap facilitates discrimination between chemical environments and, coupled with 2D correlation methods, increases the ease of structure assignment (Supporting Information, Table S4).

Cyanocobalamin contains multiple exchangeable amide signals, seen between 6 and 8 ppm (expansion shown in Figure 3). The triplet at 8.3 ppm (Figure 3a) is attributed to the secondary amide. Due to its low exchange rate, this signal survives in both the NOESY-presaturation PSYCHE and PSYCHE-iWG spectra, showing as a clear singlet at 8.3 ppm in the latter. As a result of restricted rotation, 12 distinct primary amide signals are observed (highlighted in orange in Figure 2). These signals have a much faster chemical exchange with the solvent and are therefore saturated by the NOESY-presaturation element (Figures 2b and 3b). WATERGATE solvent suppression is preferable here, as it enables retention of signals undergoing chemical exchange, as demonstrated in the PSYCHE-iWG spectra (Figures 2c and 3c). The primary amide proton signals are largely retained, though some signal loss is inevitable due to transverse relaxation; the sequence duration should be minimized if *T*_2_ relaxation is a limiting factor. Because PSYCHE-iWG does not require a long recovery delay for presaturation, it allows more scans to be acquired per unit time than NOESY-presaturation PSYCHE. Setting the initial excitation pulse to the Ernst angle,^28^ it is possible to boost the SNR of the resultant PSYCHE-iWG spectrum (see Figure 3d, and Table S3 in the Supporting Information).

### Atropine Eye-Drop Solution

**Figure 4:**
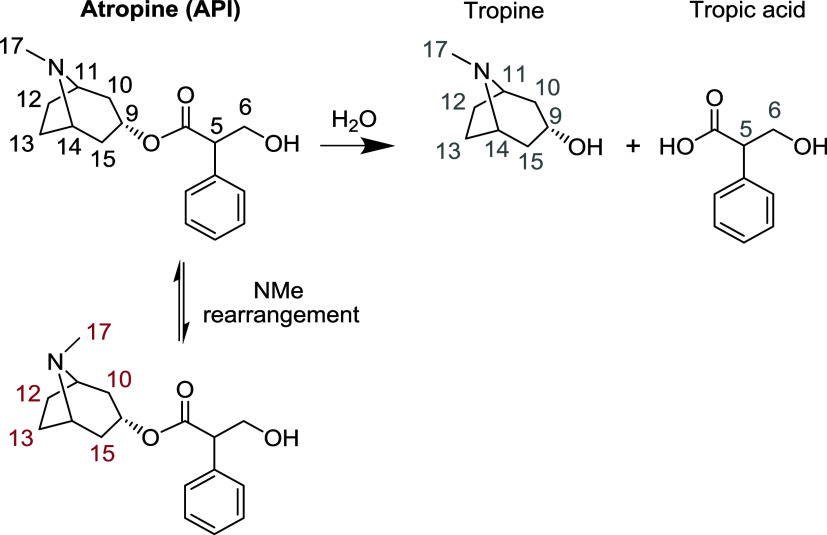
Atropine API (assignments in black) and impurities. Atropine hydrolyzes to form tropine and tropic acid (assignments in gray). A NMe rearrangement product (partial assignments in brown) is in slow exchange.

**Figure 5:**
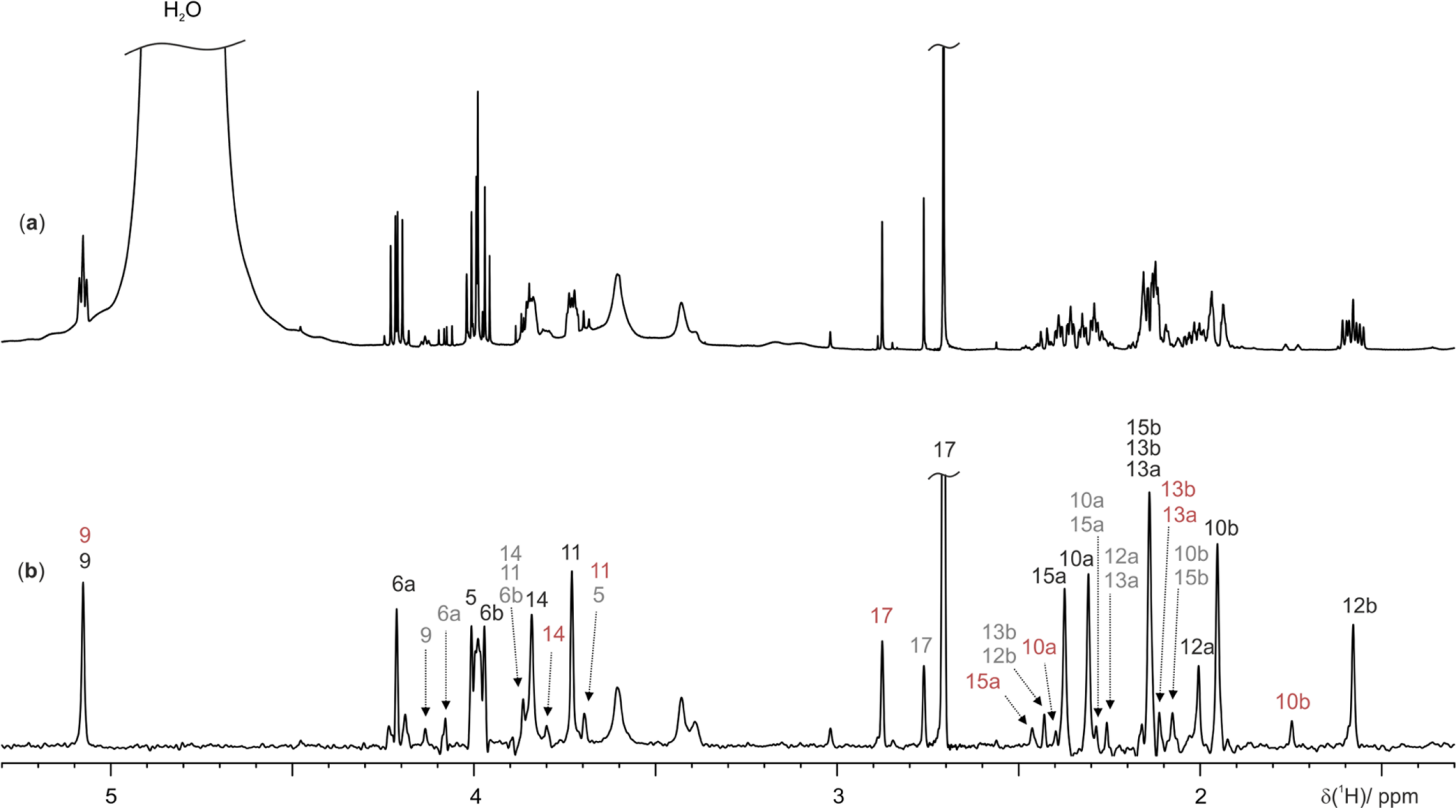
500.13 MHz ^1^H NMR spectra of an atropine eye-drop solution (90%) in D_2_O (10%), recorded at 25 °C. (a) Conventional 1D ^1^H NMR and (b) PSYCHE-iWG spectrum. Selective rectangular 90° pulses of 6 ms duration were used in the WATERGATE element. The PSYCHE element consisted of two saltire pulses of 10 kHz bandwidth, 70 ms duration, and 20° on-resonance flip angle. Sixteen chunks were acquired with a duration of 20 ms. Further experimental details are given in the Supporting Information. Atropine signal assignments are shown in black, hydrolysis products in gray, and NMe rearrangement product signals in brown.

Many pharmaceuticals are formulated in aqueous solution; the example used here, atropine eye-drops (Figure 4) for topical ocular administration, is used for conditions including myopia, cycloplegia, and amblyopia.^29,30^ The formulation contains a large concentration range of molecules, including the API, degradation products, impurities, and excipients. NMR analysis, already challenging due to the presence of multiple species with high dynamic range, is further complicated by the intense H_2_O signal (Figure 5a). The solvent suppression and ultrahigh resolution offered by the PSYCHE-iWG experiment (Figure 5b) simplify the identification of API signals and reduce signal overlap, allowing key signals from degradation products to be identified. Combining PSYCHE-iWG with 2D correlation structure elucidation methods (data not shown) enabled the characterization of the API and several impurities (Figures 4 and 5).

The significant spectral overlap in the conventional ^1^H NMR spectrum (Figure 5a) is largely avoided in the PSYCHE-iWG spectrum (Figure 5b). The WATERGATE elements completely suppress the solvent signal while retaining solute signals that are close in frequency (e.g., H9 in atropine). The API signals (in black) are immediately evident due to their higher intensity, and full assignment is provided in Figure 5b. With the improved spectral resolution observed in Figure 5b, the impurity signals, no longer eclipsed by the more intense API signals, are easier to identify. The identification of degradation products is of importance as it provides insight into the stability of the API. Hydrolysis of the ester bond cleaves atropine into tropine and tropic acid (Figure 4; peaks assigned in gray in Figure 5b). It is possible to use these signals as markers to assess the shelf life of the eye-drop solution. A further impurity, partially assigned in brown, is attributed to reversible rearrangement of the NMe group.^31^ The preferred configuration of the NMe group is equatorial (NOEs between H17 and H12, H13 prove this), and the minor configuration is axial (NOEs between H17 and H10, H15 are seen). The reversible rearrangement was confirmed by NOESY experiments, which showed exchange peaks between atropine signals and this rearrangement product (data not shown).

## Conclusions

The proposed PSYCHE-iWG pure shift method has great potential utility in the analysis of complex samples with strong solvent signals. A key benefit is the ability to retain signals undergoing chemical exchange with the solvent such as NH signals in proteins and peptides. The integration of WATERGATE directly into the pure shift pulse sequence enables the use of short recovery delays, boosting the SNR of the resultant spectrum compared with methods using presaturation.

Such sequences have further potential application in multidimensional pure shift experiments, such as TOCSY-PSYCHE,^32^ which would enable acquisition of ultrahigh-resolution correlation experiments in nondeuterated solvents. Furthermore, as PSYCHE-iWG provides chemical shift information for each multiplet, ultraselective 1D correlation NMR experiments (such as the GEMSTONE family of experiments)^33−36^ can be used as fast alternatives to 2D experiments for characterization.

## Supporting Information Available

Pulse program code; experimental details and further sample analysis (PDF)
