# Supplementary material for: Solvent Suppression in Pure Shift NMR
Source: Anal Chem. 2024 Feb 21;96(9):3879–85. doi: 10.1021/acs.analchem.3c05379 (PMC10918619; doi:10.1021/acs.analchem.3c05379)
Supplement: Supplementary file 1 — ac3c05379_si_001.pdf [file ac3c05379_si_001.pdf]

Supporting Information

Solvent suppression in pure shift NMR

Emma L. Gates,[a] Jonathan P. Bradley,[b] Daniel B. G. Berry,[b] Mathias Nilsson,[a] Gareth A. Morris,[a] Ralph W. Adams,[a] Laura Castañar*[a,c]

[a] E. L. Gates, G. A. Morris, M. Nilsson, R. W. Adams, L. Castañar*
Department of Chemistry
University of Manchester, Oxford Road, Manchester, M13 9PL, UK.

[b] J. P. Bradley, D. B. G. Berry
Johnson Matthey Technology Centre
Blounts Court Road, Sonning Common, RG4 9NH, UK.

[c] L. Castañar*
Department of Organic Chemistry, Faculty of Chemical Science
Complutense University of Madrid, 28040 Madrid, Spain.

*Corresponding author
E-mail: lcastana@ucm.es / laura.castanaracedo@manchester.ac.uk

Contents

A. Experimental details 3
1. Pulse sequence 3
2. Experimental parameters 5
B. Cyanocobalamin 8
C. Atropine eye-drop solution 14
D. Suppression profiles 17
E. Concatenated WATERGATE-PSYCHE *vs* PSYCHE-iWG 18
F. Pulse program for Bruker spectrometer 20
G. References 24

A. Experimental details

1. Pulse sequence

Further details of the PSYCHE-iWG pulse sequence, introduced in Figure 1 in the main manuscript, are provided in Figure S1. The schematic pulse sequence diagram shown in Figure S1 refers to the pulse program provided at the end of this document in Section F, coded for use with a Bruker spectrometer. The pulse program offers the user a choice of WATERGATE suppression elements, either iWG[1] (the original WATERGATE element) or iW5[2] (a binomial WATERGATE element). The user also has a choice between three active spin refocusing (ASR) elements: pure shift yielded by chirp excitation (PSYCHE),[3] Zangger-Sterk (ZS),[4] or band-selective (BS).[5,6] Discussions on the choice of both WATERGATE and ASR elements can be found in the main manuscript, with further details below.

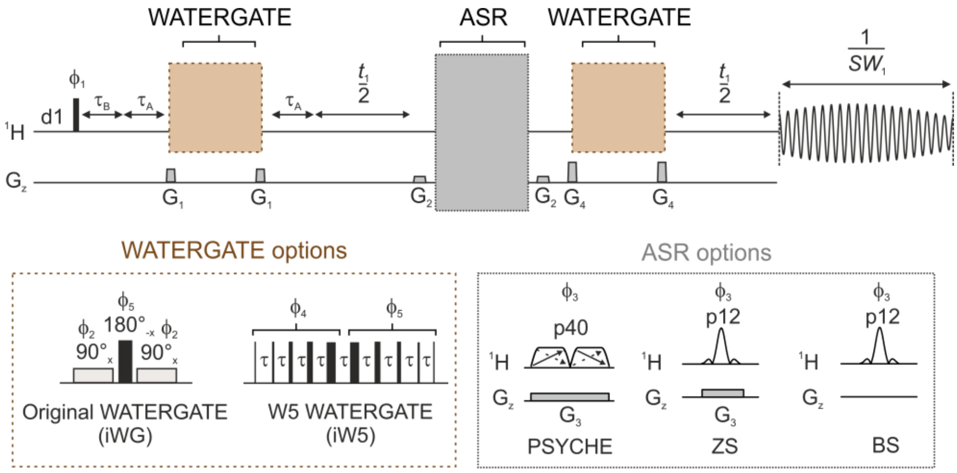

Figure S1. Schematic representation of the PSYCHE-iWG pulse sequence as implemented for Bruker instruments. Narrow, wide and short rectangles represent hard 90°, hard 180° and soft 90° radiofrequency (RF) pulses, respectively. The dashed and dotted inset boxes highlight the WATERGATE and ASR element options, respectively, available in the pulse sequence code provided. The PSYCHE ASR element is shown as trapezoids with two diagonal arrows, corresponding to low-power saltire pulses with a duration defined by p40 and a flip angle β (typically 20°). Alternatively, the BS or ZS element can be chosen; the selective bandwidth of each is controlled by the duration p12. A rectangular spatial encoding gradient pulse, G_3, is applied during the PSYCHE and ZS ASRs. The code provided has two WATERGATE element options, iWG and iW5, in which τ represents the interpulse delay of the W5 element and can be set using options in the pulse program. The experiment uses interferogram acquisition mode with incremented t_1 duration. The chunk duration is defined by $1/SW_1$. τ_A controls the time at which J-evolution is refocused during the chunk, and is set to $1/4SW_1$. τ_B represents a delay to enable an integer number of 'drop points' to be discarded from the start of the acquired data, to compensate for distortions caused by digital signal processing. The delay for magnetisation recovery is represented by d1. Shapes denoted G_1, G_2, and G_4 represent pulsed field gradients applied along the z-axis of the magnet used to reinforce the coherence transfer pathway.

The suppression bandwidth for iWG is controlled by the rectangular 90° selective pulses in the sequence. The width of the suppression region is inversely proportional to the RF pulse duration, with a narrower bandwidth requiring a longer RF pulse. The user should keep these pulses as short as possible to avoid excessive T_2 relaxation, but long enough to ensure that solute signals neighbouring the solvent signal are not suppressed. Adequate solvent suppression is routinely observed relying simply on the power calculated using the Wavemaker tool, but improved suppression can be obtained by careful optimisation of the selective pulse phase and amplitude. As an alternative to iWG, the binomial iW5 WATERGATE element can be chosen. In iW5, the interpulse delay, τ, controls the suppression bandwidth. A longer interpulse delay ensures a sharper suppression profile but, unlike iWG, the binomial sequence of pulses causes additional suppression notches to be seen at offsets which are multiples of 1/τ Hz, so the user must be careful to choose a value for τ that avoids suppression of solute signals of interest. Examples of suppression profiles using iWG or iW5 are provided in Section D. Should multiple solvent signals require suppression, a frequency-modulated selective pulse can be used in iWG to suppress several signals or, for iW5, the interpulse delay can be chosen to exploit suppression

notches at more than one frequency. Coherence transfer pathway (CTP) pulsed field gradients (PFGs) G_1 and G_4 are responsible for dephasing the on-resonance signal; the (solvent) signal that the user wishes to suppress is placed on resonance. As the sequence combines two WG elements with PFGs, excitation sculpting[7] is incorporated into the sequence, providing increased dephasing, and therefore suppression, of the solvent signal.

For acquisition of a broadband spectrum, either a PSYCHE or ZS ASR element should be used. The BIRD-based[8] pure shift approach has intrinsic water suppression due to the requirement for a ^{13}C filter. A BS ASR element provides high sensitivity spectra but is limited in spectral bandwidth as the selective pulse must not excite coupled spins. The PSYCHE and ZS ASR elements utilise a rectangular PFG (G_3) for spatial encoding, in combination with either saltire pulses or a band-selective pulse, respectively. CTP selection is performed by applying PFGs (G_2) either side of the ASR element.

The WATERGATE pure shift experiment is a pseudo-2D method using interferogram acquisition mode. Incremented evolution times are represented by $t_1/2$ periods. To provide the desired homonuclear broadband decoupling, short bursts, or chunks, are extracted from the start of each acquired FID, where *J* evolution is negligible, which are then concatenated to produce the pure shift FID. For that, the chunk duration, $1/SW_1$, must be much shorter than the inverse of the largest scalar coupling the user wishes to decouple; $1/SW_1 \ll 1/J_{HH}$. Typically, 10 - 20 ms for ^{1}H-^{1}H homodecoupling is sufficient. To ensure *J*-evolution is refocused at the midpoint of the chunk, at $1/2SW_1$, τ_A is set to $1/4SW_1$.

Table S1 and S2 show the phase cycles for the PSYCHE-iWG, ZS-iWG, BS-iWG experiments, and PSYCHE-iW5, ZS-iW5, and BS-iW5 experiments, respectively.

Table S1. Phase cycle for PSYCHE-iWG, ZS-iWG, and BS-iWG.

Coherence	Pulse phase
ϕ_1	$x_4, y_4, -x_4, -y_4$
ϕ_2	x
ϕ_3	$x, y, -x, -y$
ϕ_5	$-x$
ϕ_{rec}	$x, -x, x, -x, -y, y, -y, y, -x, x, -x, x, y, -y, y, -y$

Table S2. Phase cycle for PSYCHE-iW5, ZS-iW5, and BS-iW5.

Coherence	Pulse phase
ϕ_1	$x_4, y_4, -x_4, -y_4$
ϕ_3	$x, y, -x, -y$
ϕ_4	x
ϕ_5	$-x$
ϕ_{rec}	$x, -x, x, -x, -y, y, -y, y, -x, x, -x, x, y, -y, y, -y$

2. Experimental parameters

All raw NMR data, and Bruker pulse programs and macros used in this paper are freely available for download from DOI: 10.48420/24600114.

General acquisition and processing parameters

All spectra were recorded on a Bruker Avance NEO 500 MHz NMR spectrometer with a 5 mm room temperature triaxial gradient TBI probe with a maximum nominal z gradient strength of 67 G cm^{-1} (G_x and G_y gradients were not used here), and processed using the Bruker Topspin software package (version 4.1.4).

PSYCHE spectra shown in the main manuscript and ESI were acquired with the following parameters unless otherwise stated. Sixteen increments of 1024 complex data points were acquired with a spectral width of 10 kHz (20 ppm) and a recovery delay of 3 s, and the first 200 complex data points of each free induction decay were taken to construct the pure shift interferogram. The Gaussian window function $\exp^{-at+bt^2}$, where $a = \pi\, LB$ and $b = \frac{a}{2\, GB\, AQ}$, was used for spectral processing.

The PSYCHE ASR element used a saltire pulse bandwidth of 10 kHz, a flip angle of 20°, a duration of 30 ms, and a simultaneously applied PFG (G_3) of 1.5 G cm^{-1}. CTP gradient pulses G_1, G_2 and G_4 were set to 23.5, 31.5, and 52.9 G cm^{-1}, respectively. If convection and diffusion losses dominate spectral sensitivity, the G_2 CTP gradient should be reduced.

All pure shift 1D FIDs were constructed from the acquired pseudo-2D data using the 'pshift' macro, available at https://nmr.chemistry.manchester.ac.uk.

Cyanocobalamin

A sample of 5 mM cyanocobalamin, purchased from Sigma Aldrich, was prepared by dissolving 4.8 mg in 700 μL 90:10 H_2O:D_2O, buffered to pH 8.1. The buffered solution was prepared using 8.8 mg monobasic potassium phosphate and 162.1 mg dibasic potassium phosphate and made up to 10 mL in deionised H_2O. All spectra were acquired at 12 °C. The optimal ^{1}H hard 90° pulse duration was measured as 14.2 μs and this value used in all experiments unless otherwise stated. Spectra were referenced to sodium trimethylsilylpropionate-d_4 (TSP) at 0 ppm.

The conventional ^{1}H pulse-acquire NMR spectrum was collected with 8 scans and 4 dummy scans, a spectral width of 10 kHz (20 ppm), a recovery delay of 3 s, 65536 data points, and an acquisition time of 3.3 s. The FID was zero-filled to produce a spectrum comprising 131072 real points which was processed using Gaussian weighting (LB = -0.01 Hz and GB = 0.002). The reconstructed 1D PSYCHE FIDs were zero-filled to yield a spectrum comprising 32768 real points which was processed using Gaussian weighting (LB = -0.01 Hz and GB = 0.002).

The PSYCHE-iWG spectrum shown in the main manuscript (Figure 2c) was acquired using the pulse sequence shown in Figure 1 of the main manuscript with 64 scans and 4 dummy scans. The rectangular selective ^{1}H 90° pulses used in the WG element had a duration of 5.5 ms each and were applied at resonance with the solvent signal. The PSYCHE-iWG spectra presented in Figure 3c and in the ESI (Figures S2b and S4f) differ only in the number of scans acquired (32 instead of 64). Figure 3d demonstrates the effects of manipulating the initial excitation flip angle while increasing the number of scans and decreasing the recovery delay; 128 scans and a recovery delay of 0.5 s were used with an excitation pulse flip angle of 73° instead of 90°. The PSYCHE-iW5 spectra shown in the ESI (Figures S2c and S2d) were acquired using the pulse sequence shown in Figure S1 (using the iW5 option) with 32 scans and 4 dummy scans. The interpulse delays, τ, used in the binomial W5 element were set to 384 and 1176 μs for Figures S2c and S2d, respectively.

Comparisons to other solvent suppression pure shift methods are made in the main manuscript and the ESI. The schematic pulse sequence diagrams for each experiment are shown in Figure S3. The NOESY-presaturation PSYCHE spectrum (Figure 2b) was acquired using the pulse sequence shown in Figure S3c with a continuous wave presaturation period (τ_1) of 3 s with a B_{1max} field strength of 80 Hz applied on resonance with the solvent signal. The NOESY mixing time (τ_2) was set to 100 ms, with continuous wave saturation of the same strength applied throughout. The PFGs G_1 and G_2 were set as suggested by Bertho *et. al.*.[9] For Figure 2b, 64 scans were acquired, for Figure 3b and S4e, 32 scans were acquired. Figure S4 showed further comparisons to ES-PSYCHE,[3] presaturation PSYCHE, and concatenated W5$_n$-TSE-PSYCHE (where *n* represents the number of WG/W5 elements).[10,11] The ES-PSYCHE spectrum (Figure S4b) was acquired using the pulse sequence shown in Figure S3a with a 3 ms 180° shaped sinc with an amplitude of 283 Hz applied on resonance with the solvent signal. The presaturated PSYCHE spectrum (Figure S4d) was acquired using the pulse sequence shown in Figure S3b using a continuous wave presaturation period of 3 s with a B_{1max} field strength of 80 Hz applied on resonance with the solvent signal. The concatenated W5$_n$-TSE-PSYCHE spectrum (Figure S4c) was acquired using the pulse sequence shown in Figure S3d using two W5 elements (n = 2) to suppress the solvent, with an interpulse delay (τ) of 384 μs. It should be noted that the concatenated W5$_n$-TSE-PSYCHE suffers from the same issues as PSYCHE-iW5, with suppression notches observed at $1/\tau$. For the WG$_n$-TSE-PSYCHE, two additional swept-frequency chirp pulses with opposite direction sweeps and simultaneous gradients surround the PSYCHE ASR; 10 kHz bandwidth, 40 ms chirp pulses with a field strength of 447 Hz and gradient of 1.4 G cm^{-1} (G_5 and G_6) were used.

Atropine eye-drop solution

A sample of commercially-available atropine eye-drop solution was prepared by adding 70 μL D_2O to 630 μL solution. All NMR spectra were acquired at 25 °C with a hard ^{1}H 90° pulse duration of 15.2 μs unless otherwise stated, and referenced to TSP at 0 ppm.

The conventional ^{1}H pulse-acquire NMR spectrum used 8 scans and 4 dummy scans, a spectral width of 10 kHz (20 ppm), a recovery delay of 3 s, 65536 data points, and an acquisition time of 3.3 s. The FID was zero-filled to yield a spectrum comprising 131072 real points which was processed using Gaussian weighting (LB = -0.01 Hz and GB = 0.001).

The PSYCHE ASR element used a relatively saltire pulse duration (70 ms) to reduce artefacts arising from strong coupling. 32 scans and 4 dummy scans were acquired for each PSYCHE spectrum. The reconstructed 1D PSYCHE FIDs were zero-filled to yield a spectrum comprising 32768 real points which was processed using Gaussian weighting (LB = -0.01 Hz and GB = 0.001).

The PSYCHE-iWG spectrum shown in the main manuscript (Figure 5) was acquired using the pulse sequence shown in Figure 1 of the main manuscript, with rectangular selective ^{1}H 90° pulses in the WG element with a duration of 6 ms each. The selective pulse power and phase were optimised to achieve the best solvent suppression; a small phase shift of 2° was required for the rectangular ^{1}H 90° selective pulses. The power and phase of the shaped pulses was optimised by an arrayed experiment on the 1D ^{1}H WG experiment (data not shown). The PSYCHE-iW5 (Figure S5c) was acquired using the pulse sequence shown in Figure S1 (where iW5 was selected) with an interpulse delay (τ) of 500 μs.

The NOESY-presaturation PSYCHE spectrum shown in Figure S6d was acquired using a continuous wave presaturation period of 3 s with a B_{1max} field strength of 100 Hz. The NOESY mixing time was set to 50 ms, during which continuous wave saturation of the same strength was applied. ES-PSYCHE utilised 3 ms shaped sinc pulses with an amplitude of 283 Hz for solvent suppression. Presaturation PSYCHE used a continuous wave presaturation period of 3 s with a B_{1max} field strength of 100 Hz.

Figure S7 demonstrates the use of ZS and BS ASRs; the PSYCHE-iWG, ZS-iWG and BS-iWG spectra were acquired with 16 scans. The ZS-iWG spectrum was acquired using a band-selective pulse of 30 Hz with a

simultaneous G_3 gradient strength of 0.3 G cm^{-1}. The BS-iWG spectrum was acquired using a band-selective pulse of 50 Hz bandwidth.

Doped water sample (Supporting Information only)

A sample of D_2O doped with 1.6 mM $CuSO_4$ was prepared; the residual H_2O signal was used to plot the suppression profiles for the PSYCHE-iWG and PSYCHE-iW5 experiments (Figure S8). All spectra were acquired at 25 °C with a hard ^{1}H 90° pulse duration of 13.8 μs unless otherwise stated.

Ten increments of 1024 complex data points were acquired with a spectral width of 10 kHz (20 ppm) and a recovery delay of 5 s, and the first 200 complex data points of each free induction decay were taken to construct the pure shift interferogram.

A saltire pulse was used as the PSYCHE ASR element which had a bandwidth of 10 kHz, a flip angle of 20°, a duration of 30 ms, and a simultaneously applied PFG (G_3) of 1.5 G cm^{-1}. CTP pulse gradients G_1, G_2 and G_4 were set to 23.5, 31.5, and 52.9 G cm^{-1}, respectively. 4 scans and 4 dummy scans were acquired for each PSYCHE spectrum. The reconstructed 1D PSYCHE FIDs were zero-filled to yield a spectrum comprising 32768 real points which was processed using Gaussian weighting (LB = -0.01 Hz and GB = 0.0008). For specific WATERGATE parameters, see figure caption.

Ethanol (Supporting Information only)

A sample of 200 mM ethanol was prepared in 700 μL 90:10 H_2O:D_2O. All spectra in Figure S9 were acquired at 25 °C with a ^{1}H 90° pulse duration of 13.1 μs unless otherwise stated, and referenced to TSP at 0 ppm.

The conventional ^{1}H pulse-acquire spectrum was acquired with 8 scans and 4 dummy scans, a spectral width of 10 kHz (20 ppm), a recovery delay of 3 s, 65536 data points, and an acquisition time of 3.3 s. The FID was zero-filled to yield a spectrum comprising 131072 real points which was processed using Gaussian weighting (LB = -0.01 Hz and GB = 0.001).

The PSYCHE-iWG and concatenated WG_n-TSE-PSYCHE shown in Figure S9 were acquired with rectangular selective ^{1}H 90° pulses in the WG element of 4 ms duration each. For the WG_n-TSE-PSYCHE, two additional swept-frequency chirp pulses with opposite direction sweeps and simultaneous gradients surround the PSYCHE ASR; 10 kHz bandwidth, 40 ms chirp pulses with a field strength of 447 Hz and an encoding gradient G_3 of 1.4 G cm^{-1} were used. Each reconstructed 1D PSYCHE FID was zero-filled to produce a spectrum comprising 32768 real points which was processed using a Gaussian weighting (LB = -0.01 Hz and GB = 0.001).

B. Cyanocobalamin

A comparison of the proposed PSYCHE-iWG and PSYCHE-iW5 approaches is shown in Figure S2.

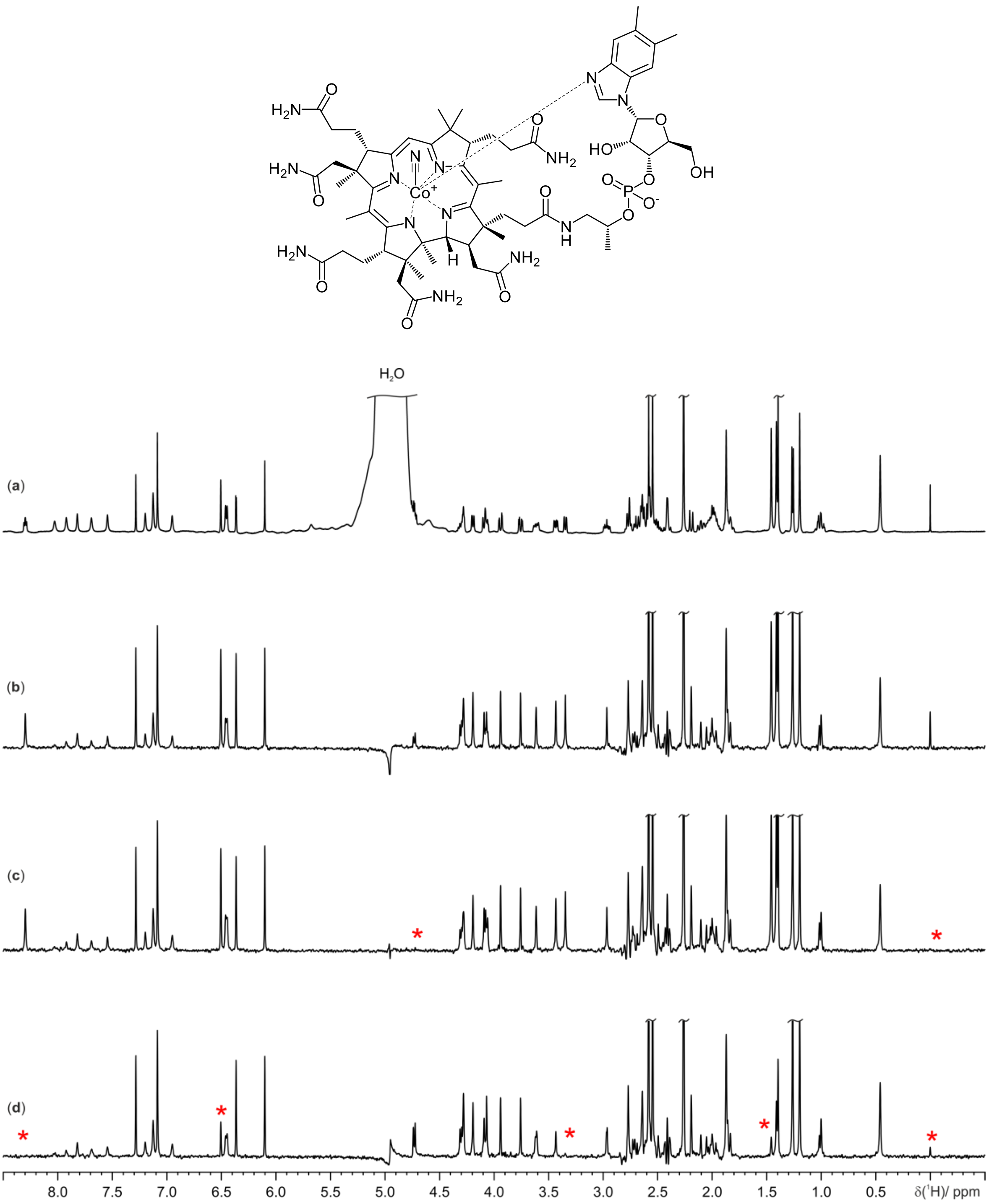

Figure S2: 500 MHz ^{1}H NMR spectra of 5 mM cyanocobalamin in 90:10 H_2O:D_2O at 12 °C and pH 8.1. (a) Conventional 1D ^{1}H NMR spectrum. (b) PSYCHE-iWG spectrum using 5.5 ms rectangular selective pulses in the WATERGATE element. (c) PSYCHE-iW5 spectrum using an interpulse delay, τ, of 384 μs in the W5 WATERGATE element. (d) PSYCHE-iW5 spectrum using an interpulse delay, τ, of 1176 μs in the W5 WATERGATE element. The PSYCHE element consisted of two consecutive saltire pulses of 10 kHz bandwidth and 30 ms duration, calibrated to achieve a 20° on-resonance flip angle. A simultaneous spatial encoding gradient, G_3, of 1.5 G cm^{-1} was applied. 16 chunks were acquired with durations of 20 ms. Red asterisks indicate fully or partially attenuated solute signals as a result of the suppression notches that the W5 sequence generates.

Cyanocobalamin has a complex ^{1}H NMR spectrum (Figure S2a) with proton signals near the solvent peak (~100 Hz in Figure S2), so a narrow suppression band is required. For PSYCHE-iWG, the rectangular selective

^{1}H 90° pulses used were extended in duration to retain these solute signals (Figure S2b). For PSYCHE-iW5, a short interpulse delay τ in the binomial W5 sequence resulted in a broader suppression profile causing suppression of the solute signal at 4.74 ppm, marked by a red asterisk (Figure S2c). A sharper suppression profile was achieved by increasing the interpulse delay τ in the binomial W5 sequence, but this causes suppression notches to be observed throughout the spectrum and, as the proton signals span 8 ppm, the loss of solute signals at 8.30, 6.51, 3.36, and 1.46 ppm, highlighted with red asterisks (Figure S2d). It should be noted that if solute signals are suppressed, not only will these be absent from the pure shift spectrum, but the effects of their couplings to other spins will not be suppressed. Although iW5 is arguably easier to set up, it is recommended that iWG is used rather than iW5 to ensure the retention of all solute signals. Further comparisons of the PSYCHE-iWG and PSYCHE-iW5 suppression profiles can be found in Section D.

A comparison of several solvent suppression pure shift methods is provided in Figure S4; the schematic pulse sequences can be found in Figure S3.

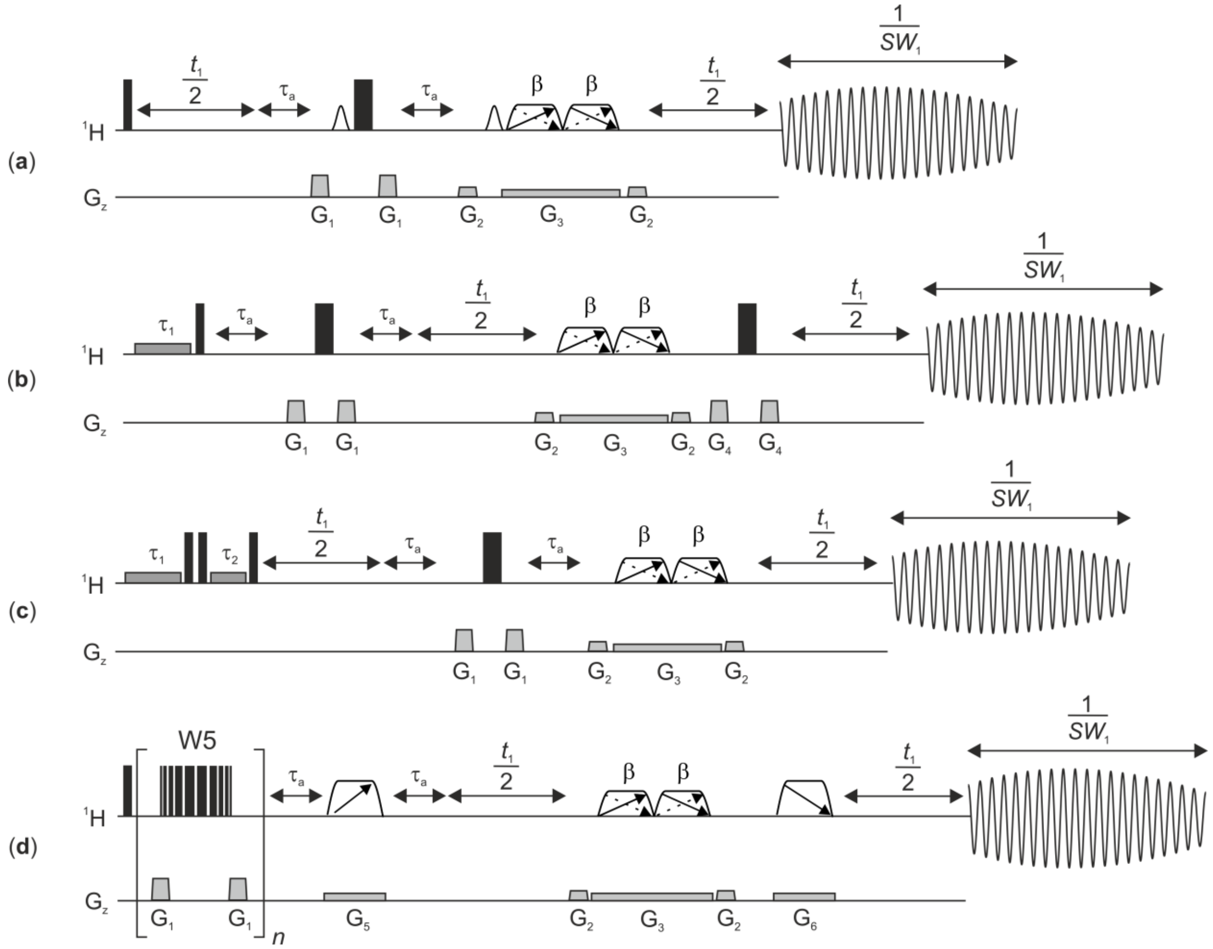

Figure S3: Schematic representations of alternative pure shift solvent suppression pulse sequences to PSYCHE-iWG. Narrow and wide rectangles represent hard 90° and 180° RF pulses, respectively. The PSYCHE ASR element is represented by trapezoids with two diagonal arrows corresponding to low-power saltire pulses with defined flip angle of β (typically 20°). A rectangular spatial encoding gradient pulse, G_3, is applied during the PSYCHE element. The trapezoids with unidirectional arrows represent adiabatic 180° chirp pulses. The interferogram acquisition mode uses incremented t_1 duration. The chunk duration is defined by $1/SW_1$. τ_A controls the time at which J evolution is refocused during the chunk, and is set to $1/4SW_1$. Shapes denoted G_1, G_2, and G_4 represent CTP pulsed field gradients applied along z. G_5 and G_6 are spatial encoding gradients. (**a**) ES-PSYCHE (excitation sculpting PSYCHE) pulse sequence.[3] Shaped sinc pulses of 3 ms duration with an amplitude of 283 Hz are used for selective water refocusing. (**b**) Presaturation-TSE-PSYCHE pulse sequence. The presaturation period τ_1 is during the recovery delay (d1). For comparison purposes, hard 180° pulses were used instead of chirp swept-frequency pulses in the TSE-PSYCHE. (**c**) NOESY-presaturation PSYCHE sequence.[9] τ_2 represents the NOESY mixing time period during which continuous solvent irradiation is applied. (**d**) Concatenated $W5_n$-TSE-PSYCHE pulse sequence.[10] The W5 sequence is comprised of a series of hard pulses with a time τ between pulses.

Excitation-sculpting PSYCHE (ES-PSYCHE),[3] presaturation PSYCHE, NOESY-presaturation PSYCHE,[9] and concatenated W5$_n$-TSE-PSYCHE,[10] (Figures S3a, S3b, S3c and S3d, respectively) are all compared with the new PSYCHE-iWG method (Figure S1) for cyanocobalamin in Figure S4.

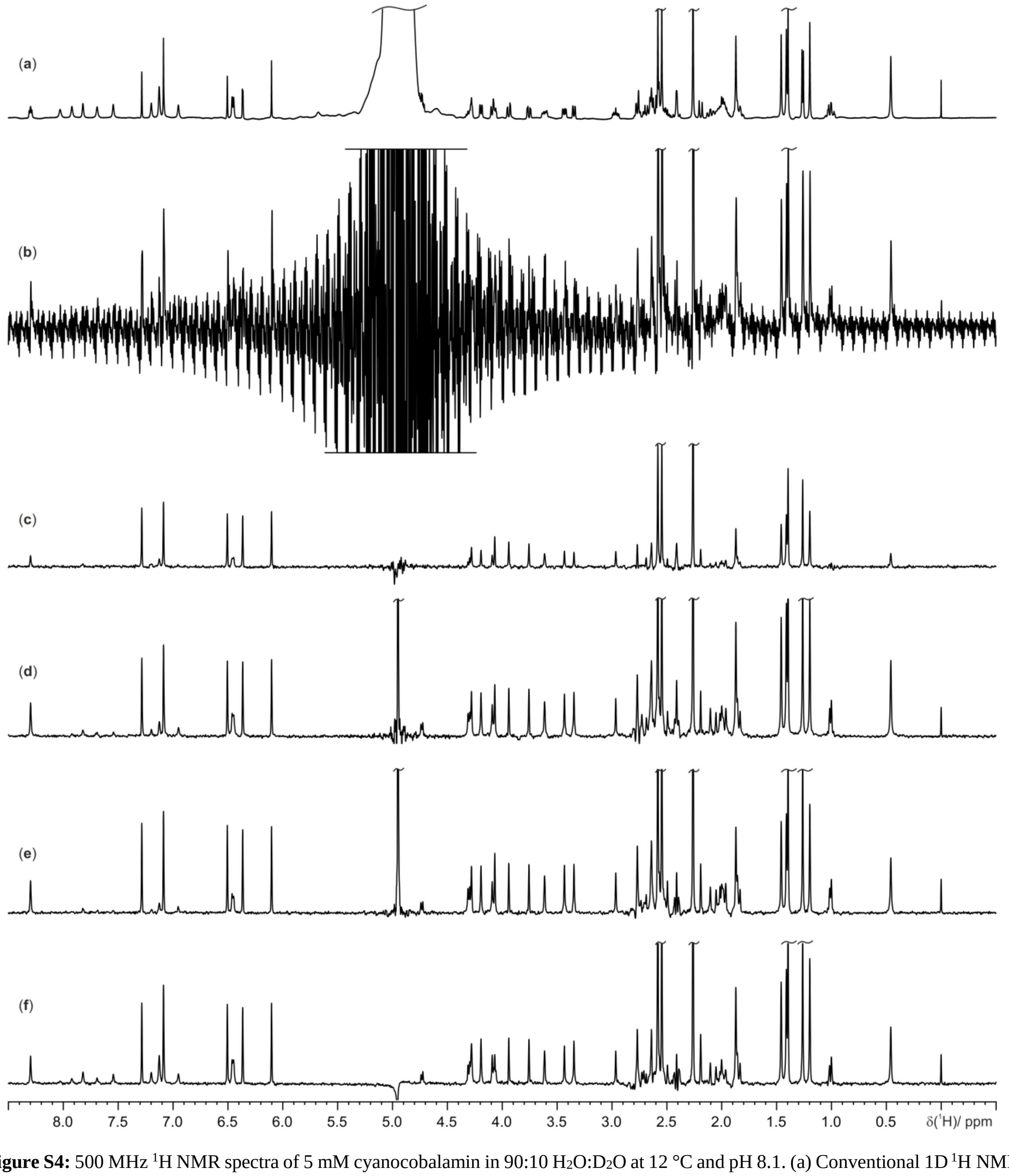

Figure S4: 500 MHz ^{1}H NMR spectra of 5 mM cyanocobalamin in 90:10 H_2O:D_2O at 12 °C and pH 8.1. (a) Conventional 1D ^{1}H NMR spectrum. (b) ES-PSYCHE spectrum using 3 ms sinc 180° with an amplitude of 283 Hz pulses for solvent suppression. (c) Concatenated W5$_2$-PSYCHE spectrum using an interpulse delay τ of 384 μs in the W5 WATERGATE element. (d) Presaturation PSYCHE spectrum with a presaturation period τ_1 of 3 s (B_{1max} field strength of 80 Hz). (e) NOESY-presaturation PSYCHE spectrum with a presaturation period τ_1 of 3 s and a NOESY mixing period τ_2 of 100 ms. (f) PSYCHE-iWG spectrum using 5.5 ms rectangular selective pulses in the WATERGATE element. In all experiments, the PSYCHE element consisted of two consecutive saltire pulses of 10 kHz bandwidth and 30 ms duration, calibrated to achieve a 20° on-resonance flip angle. A simultaneous spatial encoding gradient, G_3, of 1.5 G cm^{-1} was applied. 16 chunks were acquired, each of 20 ms duration.

ES-PSYCHE (Figure S4b) does not provide sufficient solvent suppression, resulting in large artefacts originating from the solvent signal spanning the pure shift spectrum; similar findings are shown in the literature.[9] The other published methods show comparably good solvent suppression to PSYCHE-iWG, and each spectrum could be analysed (Figure S4c-f). The concatenated $W5_n$-TSE-PSYCHE experiment suffers from the same issue (multiple suppression bands) as PSYCHE-iW5, and care must be taken to ensure no solute signals are suppressed. Furthermore, as the $W5_n$ element is concatenated with TSE-PSYCHE (Figure S3d), *J*-evolution occurs during the $W5_n$ block, which means homonuclear scalar couplings are no longer refocused in the centre of the chunk. As a result, larger chunking sidebands can be observed, which hinder the analysis of the spectrum. It should be noted that these chunking sidebands are only observable for high signal-to-noise ratio pure shift spectra (see Section E) and are not observed in Figure S4c due to the low sensitivity of the spectrum. Both presaturation PSYCHE (Figure S4d) and NOESY-presaturation PSYCHE (Figure S4e) result in the loss, or partial loss, of signals undergoing chemical exchange with the solvent (exchangeable amide proton signals are present between 6 and 8.5 ppm). PSYCHE-iWG (Figure S4f) both retains these exchanging signals and refocuses scalar evolution during the WATERGATE elements so does not generate strong chunking sidebands (see Section E). Each suppression method offers advantages and disadvantages. WATERGATE methods have the benefit of retaining signals undergoing chemical exchange with the solvent signal, while presaturation methods typically have narrower suppression profiles.

As mentioned previously, the WG solvent suppression element is integrated directly into the new PSYCHE-iWG pulse sequence, unlike presaturation methods which rely on a low-powered, long continuous wave pulse to saturate the solvent signal, typically during the recovery delay. This means that shorter recovery delays can be used in PSYCHE-iWG than in presaturation PSYCHE experiments. Per unit time, this allows acquisition of more scans if PSYCHE-iWG is used, and with manipulation of the initial excitation pulse according to the Ernst equation,[12] a higher SNR is achieved. This is demonstrated in Figure 3 in the main manuscript, where a signal enhancement for the aromatic and amide signals in cyanocobalamin is observed when optimising the experiment according to the Ernst equation. Figure 3c (PSYCHE-iWG) and Figure 3b (NOESY-presaturation PSYCHE) were acquired using the same recovery delay, and Figure 3d (PSYCHE-iWG) was acquired using a shorter recovery delay but the same experiment time as Figures 3c and 3b. The SNRs of the signals for each experiment are provided in Table S3. As seen from Table S3 and in Figure 3, manipulation of the PSYCHE-iWG experiment (Figure 3d) enables higher SNRs to be recorded for each signal than in the NOESY-presaturation PSYCHE experiment (Figure 3b).

Table S3: Signal-to-noise ratio (SNR) of the resolved signals between 6 and 8.5 ppm in the spectra shown in Figure 3.

δ(^{1}H)/ ppm	SNR		
	**NOESY-presat PSYCHE (Figure 2b)**	**PSYCHE-iWG (Figure 2c)**	**PSYCHE-iWG (Figure 2d)**
8.30	25	25	35
8.03	0	2	3
7.92	0	4	5
7.82	3	10	12
7.69	0	5	6
7.55	0	7	9
7.29	70	72	70
7.20	4	10	13
7.13	7	24	30
7.09	77	88	94
6.95	6	9	12
6.51	67	69	76
6.37	65	67	73
6.10	65	71	93

PSYCHE-iWG and 2D correlation experiments enabled the assignment of the protons for cyanocobalamin (Table S4). Molecular structure with the labelling used in the assignments1 is shown below.

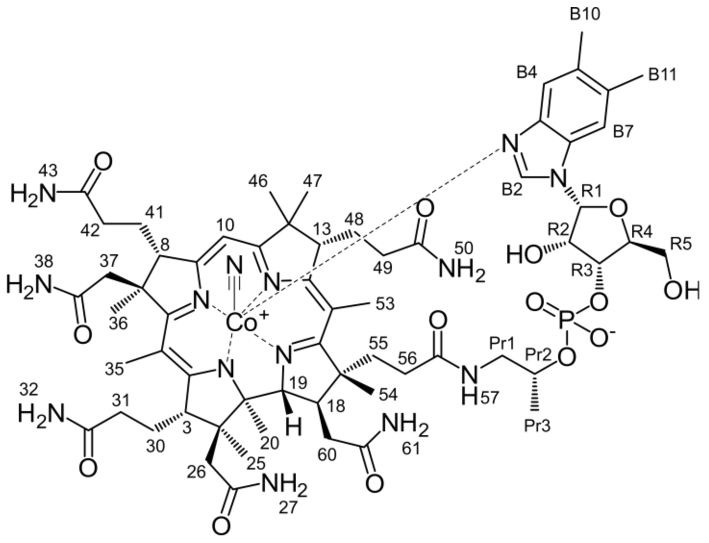

Table S4: Assignments of cyanocobalamin (pH 8.1, 12 °C, H2O (90%): D2O (10%)).

**Atom label**	**Atom type**	**δ(^{1}H) / ppm**
NH57	NH	8.30
B7	CH	7.28
B2	CH	7.09
B4	CH	6.50
NH43	NH_2	6.46
R1	CH	6.36
10	CH	6.10
R3	CH	4.73
Pr2	CH	4.30
R2	CH	4.28
3	CH	4.19
19	H	4.09
R4	CH	4.07
R5	CH_2	3.94, 3.76
Pr1	CH_2	3.62, 2.97
8	CH	3.43
13	CH	3.35
60	CH_2	2.77, 2.68
18	CH	2.76
55	CH_2	2.64,1.83
49	CH_2	2.64
37	CH_2	2.59, 2.19
53	CH_3	2.58
31	CH_2	2.57, 2.50
35	CH_3	2.55
56	CH_2	2.54, 2.10
26	CH_2	2.41
B11	CH_3	2.26
B10	CH_3	2.26
30	CH_2	2.05, 1.97
48	CH_2	2.01, 1.97
41	CH_2	2.00, 1.00
36	CH_3	1.87
42	CH_2	1.86, 1.02
47	CH_3	1.46
25	CH_3	1.41
54	CH_3	1.40
Pr3	CH_3	1.26
46	CH_3	1.20
20	CH_3	0.46
NH*	NH_2	7.69, 6.95
NH*	NH_2	7.93, 7.21
NH*	NH_2	7.55, 7.13
NH*	NH_2	7.82, 7.08
NH*	NH_2	8.03, 7.12

*Not explicitly assigned

C. Atropine eye-drop solution

Figure S5 shows a comparison between the PSYCHE-iWG and PSYCHE-iW5 methods. Partial loss of the H9 signal from atropine at 5.08 ppm is observed when PSYCHE-iW5 is used (Figure S5c); a sharper suppression profile could be obtained by increasing the interpulse delay τ, however this risks the loss of signals elsewhere in the spectrum.

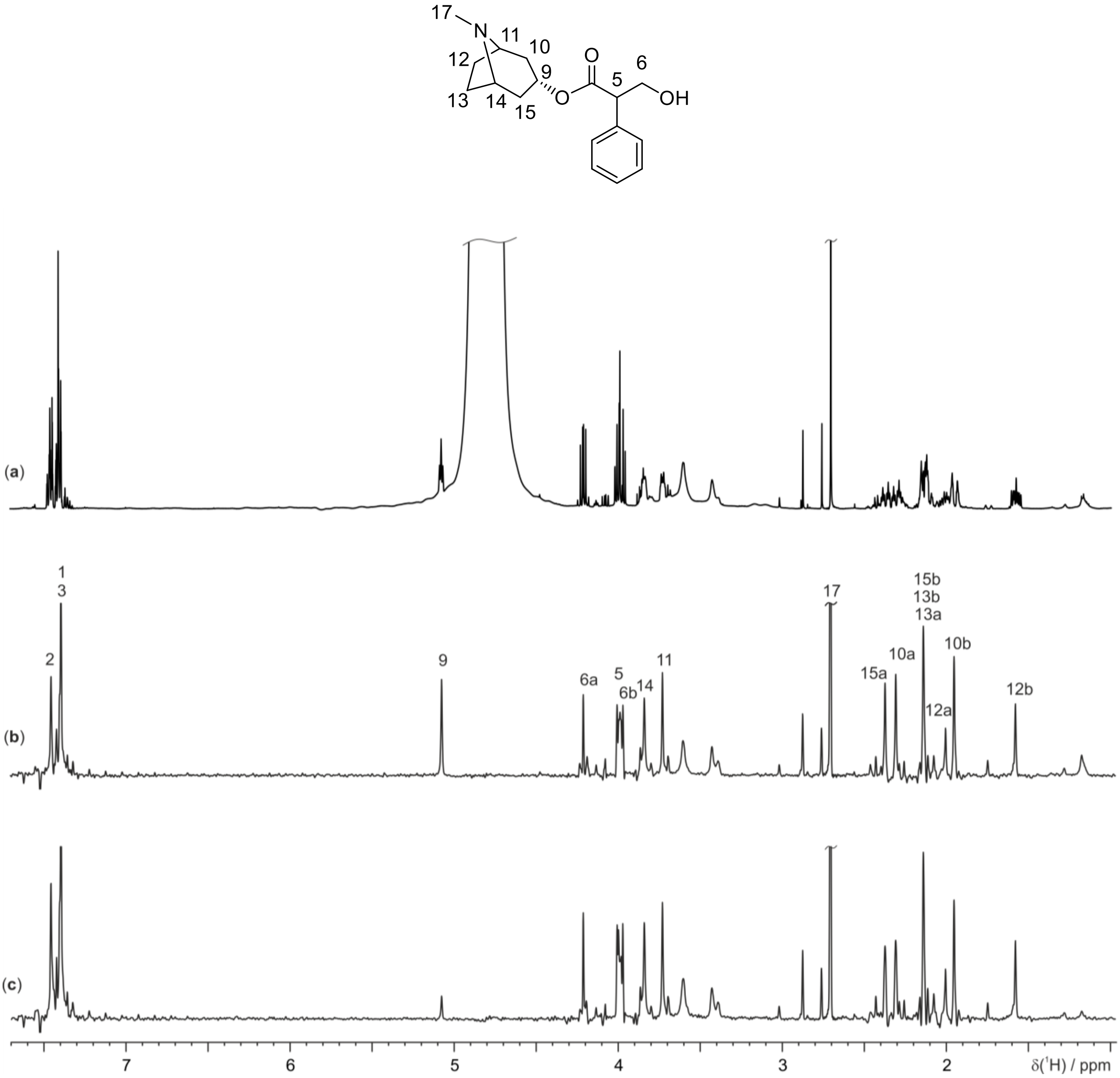

Figure S5: 500 MHz ^{1}H NMR spectra of atropine eye-drop solution in 10% D_2O recorded at 25 °C. (a) Conventional 1D ^{1}H NMR spectrum. (b) PSYCHE-iWG spectrum using 6 ms rectangular selective ^{1}H 90° pulses in the WATERGATE element. (c) PSYCHE-iW5 spectrum using an interpulse delay τ of 500 μs in the binomial W5 WATERGATE element. The PSYCHE element used two consecutive saltire pulses of 10 kHz bandwidth and 70 ms duration, calibrated to achieve a 20° on-resonance flip angle. A simultaneous spatial encoding gradient, G_3, of 1.5 G cm^{-1} was applied. 16 chunks were acquired, each of 20 ms duration.

Comparisons between different solvent suppression pure shift methods for the atropine eye-drop sample are shown in Figure S6. Again, the ES-PSYCHE (Figure S6b) shows poor solvent suppression whereas the presaturation PSYCHE (Figure S6c), NOESY-presaturation PSYCHE (Figure S6d), and PSYCHE-iWG (Figure S6e) show comparatively good solvent signal suppression, with PSYCHE-iWG showing effectively complete solvent suppression.

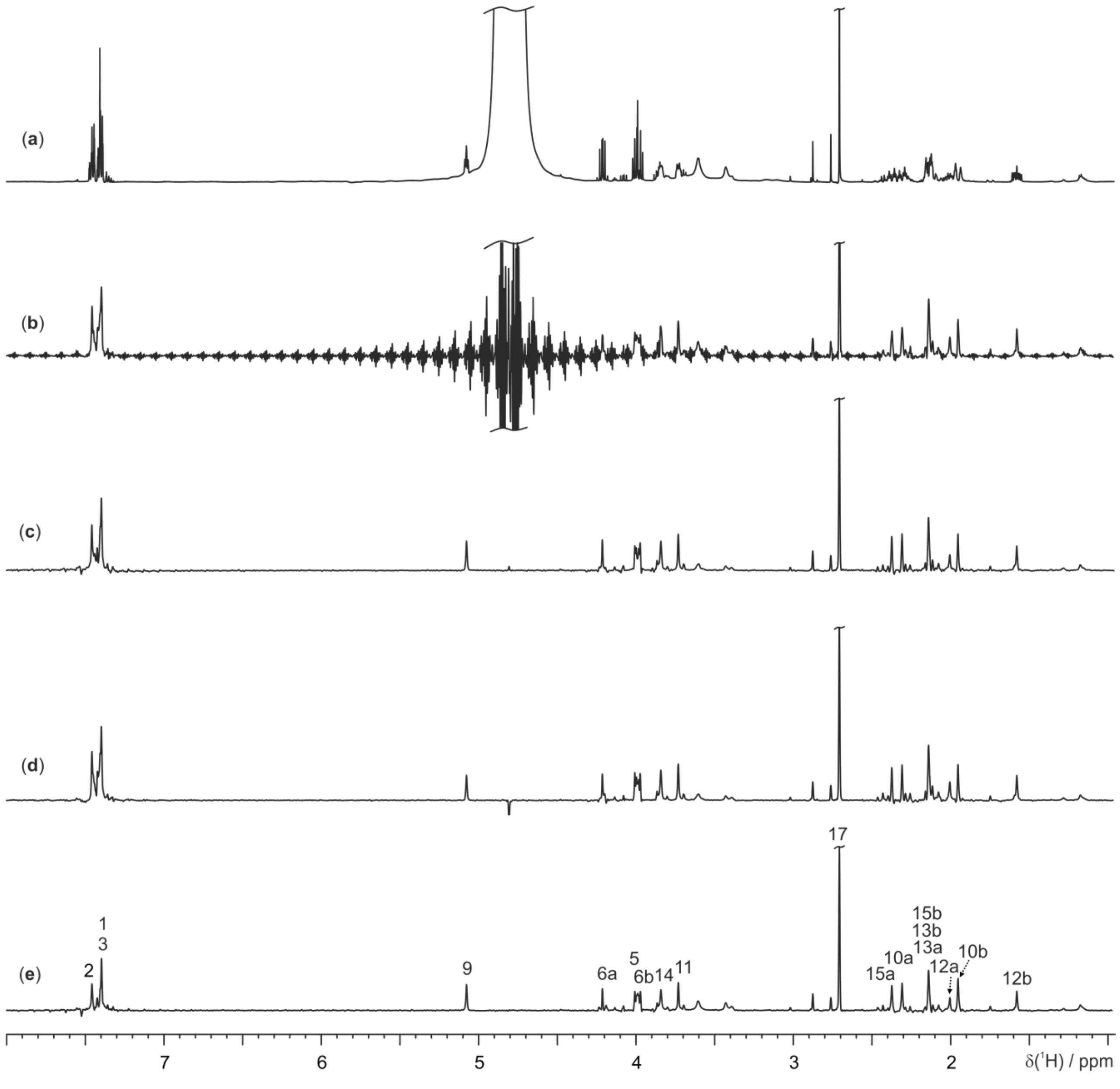

Figure S6: 500 MHz ^{1}H NMR spectra of atropine eye-drop solution in 10% D_2O recorded at 25 °C. (a) Conventional 1D ^{1}H NMR spectrum. (b) ES-PSYCHE spectrum using 3 ms 180° sinc pulses (with an amplitude of 283 Hz) for solvent suppression. (c) Presaturation PSYCHE spectrum with a presaturation period τ_1 of 3 s (B_{1max} field strength of 100 Hz). (d) NOESY-presaturation PSYCHE spectrum with a presaturation period τ_1 of 3 s (B_{1max} field strength of 100 Hz) and a NOESY mixing period τ_2 of 50 ms. (e) PSYCHE-iWG spectrum using 6 ms rectangular selective ^{1}H 90° pulses in the WATERGATE element. The PSYCHE element consisted of two consecutive saltire pulses of 10 kHz bandwidth and 70 ms duration, calibrated to achieve a 20° on-resonance flip angle. A simultaneous spatial encoding gradient, G_3, of 1.5 G cm^{-1} was applied. 16 chunks were acquired, each of 20 ms duration, for all pure shift experiments.

The PSYCHE ASR element can be exchanged for either BS or ZS. Example spectra acquired with these methods are shown in Figure S7. Figure S7b shows an example of a BS pure shift spectrum, where signal intensity is largely preserved. The ZS ASR element can be adjusted to cover either the full spectral width or a selected spectral region (as shown in Figure S7c). The ZS spectral sensitivity is dependent on the slice selectivity; the selective 180° pulse in combination which a spatial encoding gradient G_3 controls the slice thickness, which determines the number of nuclei contributing to spectrum.[13–16] Here reducing the bandwidth covered gives the ZS method a sensitivity almost equal to that of PSYCHE.

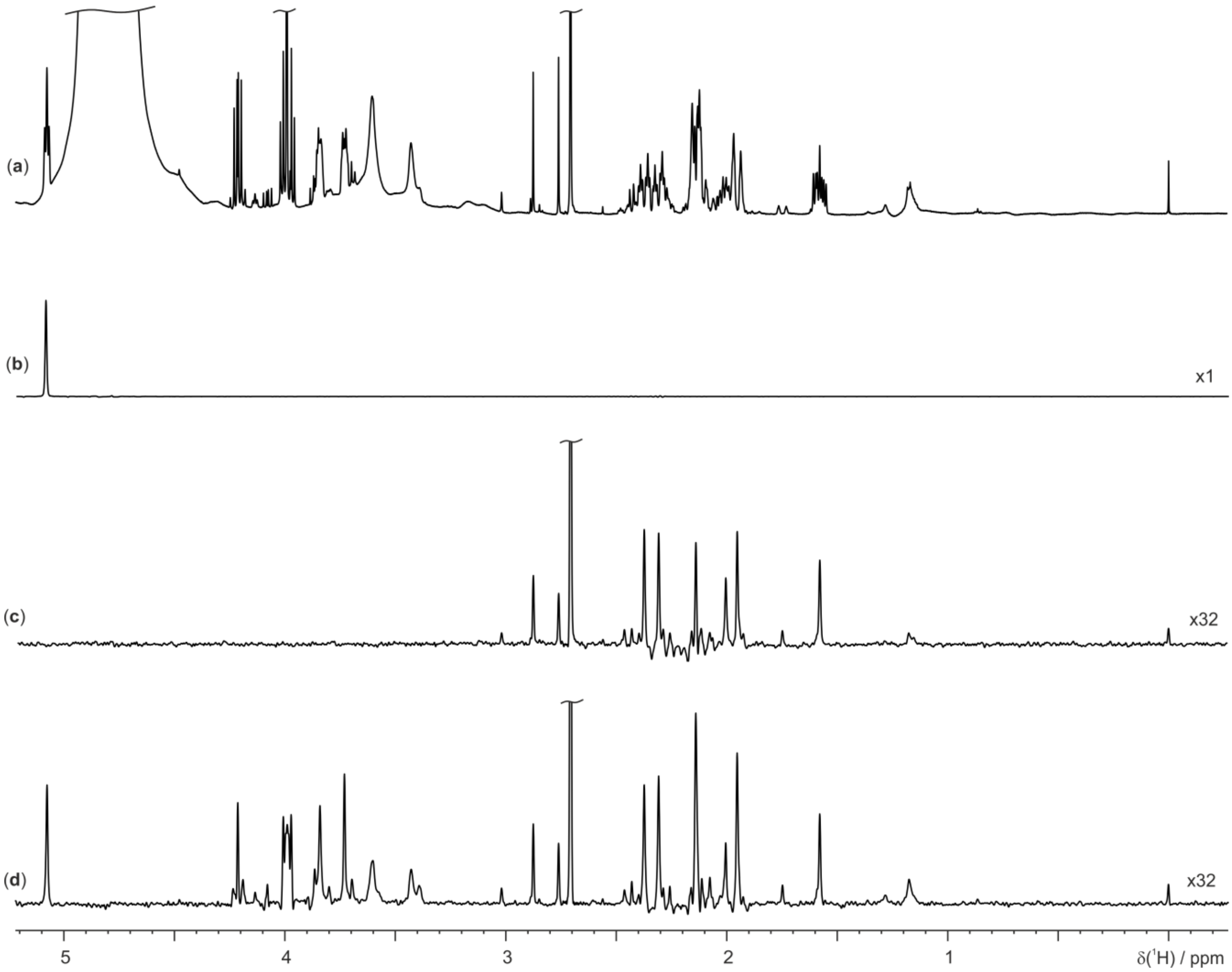

Figure S7: 500 MHz ^{1}H NMR spectra of atropine eye-drop solution in 10% D_2O recorded at 25 °C. (a) Conventional 1D ^{1}H NMR spectrum, (b) BS-iWG spectrum targeting the signal at 4.97 ppm using a band-selective 180° Rsnob pulse with a 50 Hz bandwidth. (c) ZS-iWG spectrum selectivity exciting the region between 1 and 3 ppm using a band-selective 180° Rsnob pulse of 50 Hz with a simultaneous spatially encoding gradient G_3 of 0.3 G cm^{-1}. (d) PSYCHE-iWG spectrum using a 6 ms rectangular selective 90° pulses in the WATERGATE element. The PSYCHE element consisted of two consecutive saltire pulses of 10 kHz bandwidth and 70 ms duration, calibrated to achieve a 20° on-resonance flip angle. A simultaneous spatial encoding gradient, G_3, of 1.5 G cm^{-1} was applied. 16 chunks were acquired, each of 20 ms duration, for all pure shift experiments.

D. Suppression profiles

A doped water sample was used to plot the suppression profiles of PSYCHE-iWG (Figures S8a and S8b) and PSYCHE-iW5 (Figures S8c and S8d). The profiles were recorded by arraying the frequency of targeted suppression from -0.3 to 9.7 ppm in steps of 0.2 ppm. Figures S8c and S8d shows the multiple suppression notches observed in PSYCHE-iW5, which occur at intervals of $1/\tau$. To achieve a narrow suppression profile, a long selective 90° pulse or long interpulse delay τ is required. For PSYCHE-iWG, there is little consequence to extending the duration of the selective 90° pulse other than increased time for relaxation to occur. For PSYCHE-iW5, care must be taken to ensure the suppression notches do not hit solute signals.

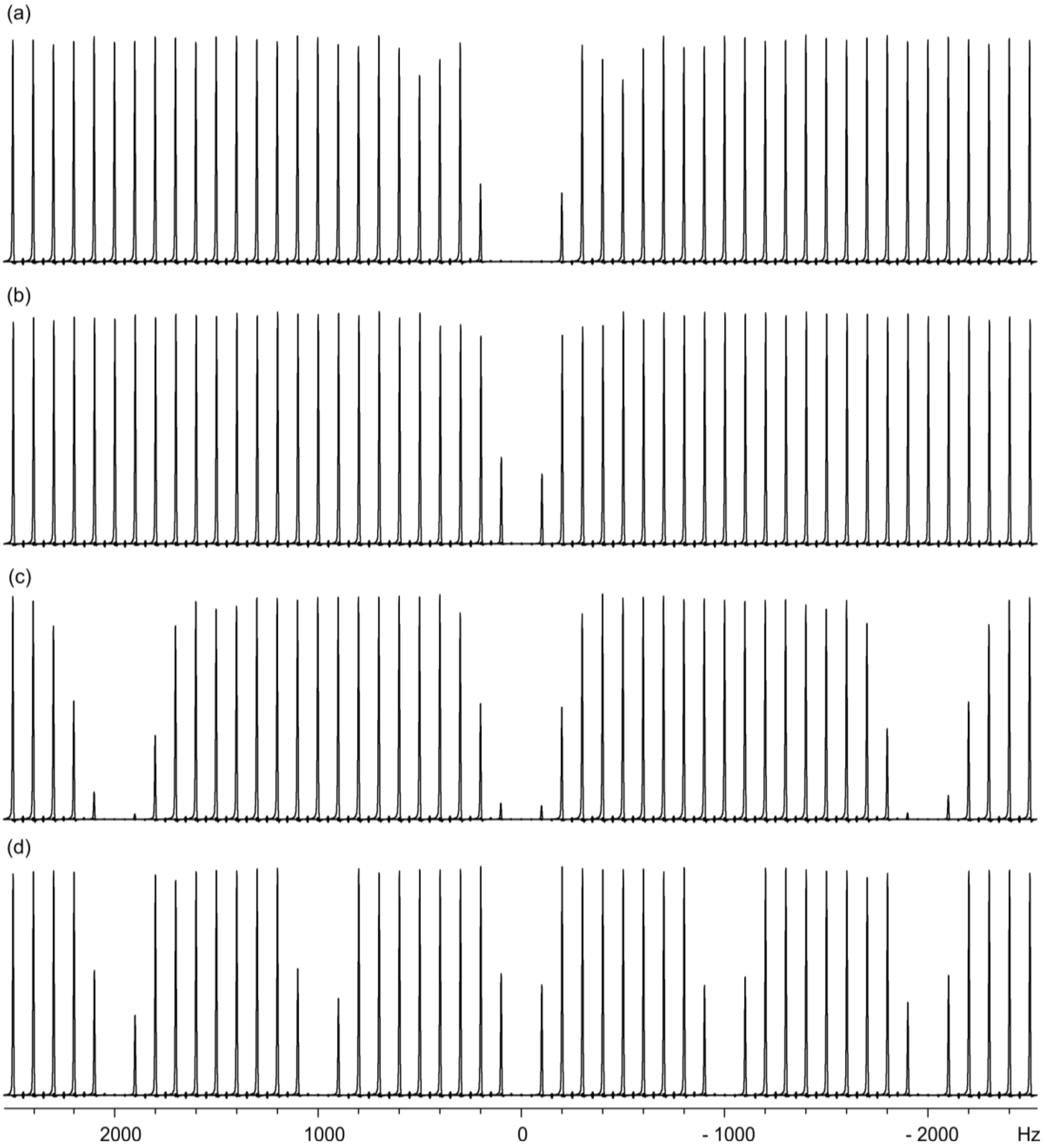

Figure S8: 500 MHz ^{1}H NMR spectra of 90:10 H_2O:D_2O doped with 1.6 mM $CuSO_4$ recorded at 25 °C. Suppression profiles of (a and b) the PSYCHE-iWG experiment using rectangular selective ^{1}H 90° pulses of 3 ms and 6 ms, respectively, and (c and d) the PSYCHE-iW5 experiment with interpulse delays τ of 500 µs and 1000 µs, respectively. The profiles were recorded by arraying the frequency of targeted suppression from -0.3 to 9.7 ppm in steps of 0.2 ppm. The PSYCHE element consisted of two consecutive saltire pulses of 10 kHz bandwidth and 30 ms duration and were calibrated to achieve a 20° on-resonance flip angle. A simultaneous spatial encoding gradient, G_3, of 1.5 G cm^{-1} was applied. 10 chunks of 20 ms duration were acquired for all pure shift experiments.

E. Concatenated WATERGATE-PSYCHE *vs* PSYCHE-iWG

The concatenated WG$_n$-TSE-PSYCHE[10] pulse sequence (shown in Figure S3d - WG used here instead of W5) is compared with the PSYCHE-iWG pulse sequence (shown in Figure S1) using a concentrated ethanol sample. WG$_1$-TSE-PSYCHE and WG$_2$-TSE-PSYCHE compared with PSYCHE-iWG in Figure S9 (utilising multiple WG elements can improve the level of solvent suppression). Chunking sidebands are evident in the spectra produced using the concatenated WG$_n$-TSE-PSYCHE sequence (Figures S9b and S9c); similar results occur when the original W5 is used rather than WG (data not shown). The presence of chunking sidebands is a consequence of additional scalar evolution during the W5/WG elements. This additional evolution is not accounted for in the WG$_n$-TSE-PSYCHE sequence, causing additional *J*-evolution during the acquisition which leads to signal discontinuities in the reconstructed data and presents as chunking sidebands in the processed spectra.

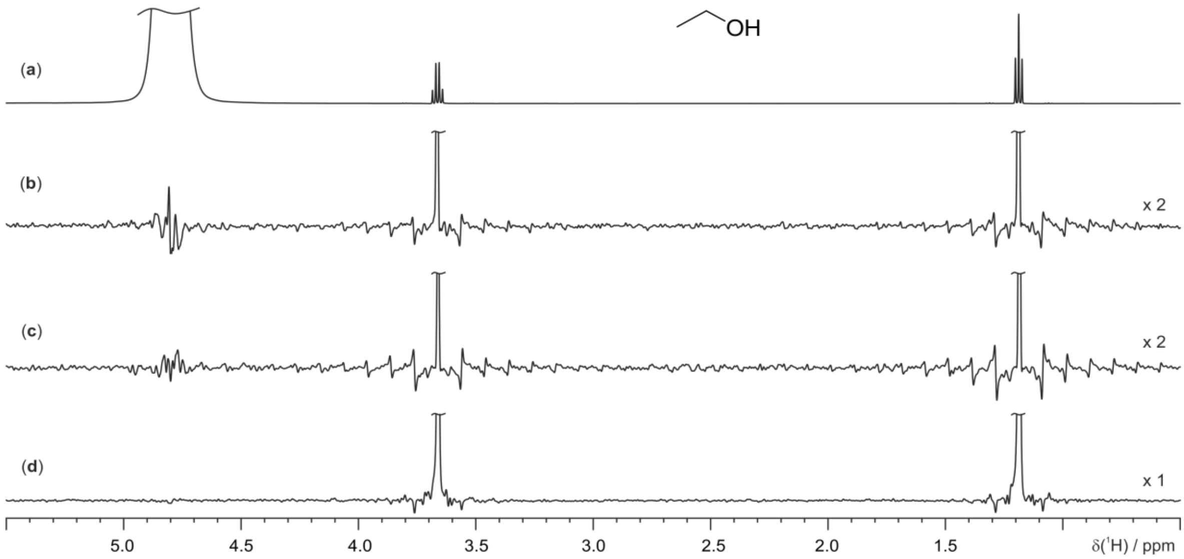

Figure S9: 500 MHz ^{1}H NMR spectra of 200 mM ethanol in 90:10 H_2O:D_2O recorded at 25 °C. (a) Conventional 1D ^{1}H NMR spectrum. (b) Concatenated WG$_1$-TSE-PSYCHE, (c) concatenated WG$_2$-TSE-PSYCHE, and (d) PSYCHE-iWG spectra using 4 ms rectangular selective ^{1}H 90° pulses in the WATERGATE element. The PSYCHE element consisted of two consecutive saltire pulses of 10 kHz bandwidth and 30 ms duration, calibrated to achieve a 20° on-resonance flip angle. A simultaneous spatial encoding gradient, G$_3$, of 1.5 G cm^{-1} was applied. 16 chunks of 20 ms duration were acquired.

As noted in the main manuscript, a perfect echo[17] adaptation of the concatenated WG$_n$/W5$_n$-TSE-PSYCHE sequence enables refocusing of the *J*-evolution during the WATERGATE elements (pulse sequence introduced in Figure S10). Simulated data (Spinach[18]) comparing the chunking sidebands from the different approaches in a simple AX spin system are shown in Figure S11.

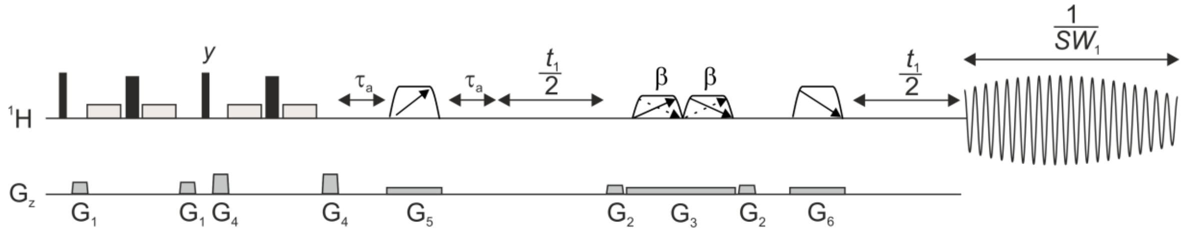

Figure S10: Schematic representation of an alternative pure shift solvent suppression pulse sequence to PSYCHE-iWG. Narrow, wide and short rectangles represent hard 90°, hard 180° and soft 90° RF pulses, respectively. The PSYCHE ASR element is represented by trapezoids with two diagonal arrows corresponding to low-power saltire pulses of defined flip angle β (typically 20°). A rectangular spatial encoding gradient pulse, G$_3$, is applied during the PSYCHE element. Trapezoids with unidirectional arrows represent adiabatic 180° chirp pulses. The interferogram acquisition mode uses incremented t_1 durations. The chunk duration is defined by $1/SW_1$. τ_A controls the time at which *J*-evolution is refocused during the chunk, and is set to $1/4SW_1$. Shapes denoted G$_1$, G$_2$, and G$_4$ represent CTP pulsed field gradients applied along *z*. G$_5$ and G$_6$ are spatial encoding gradients.

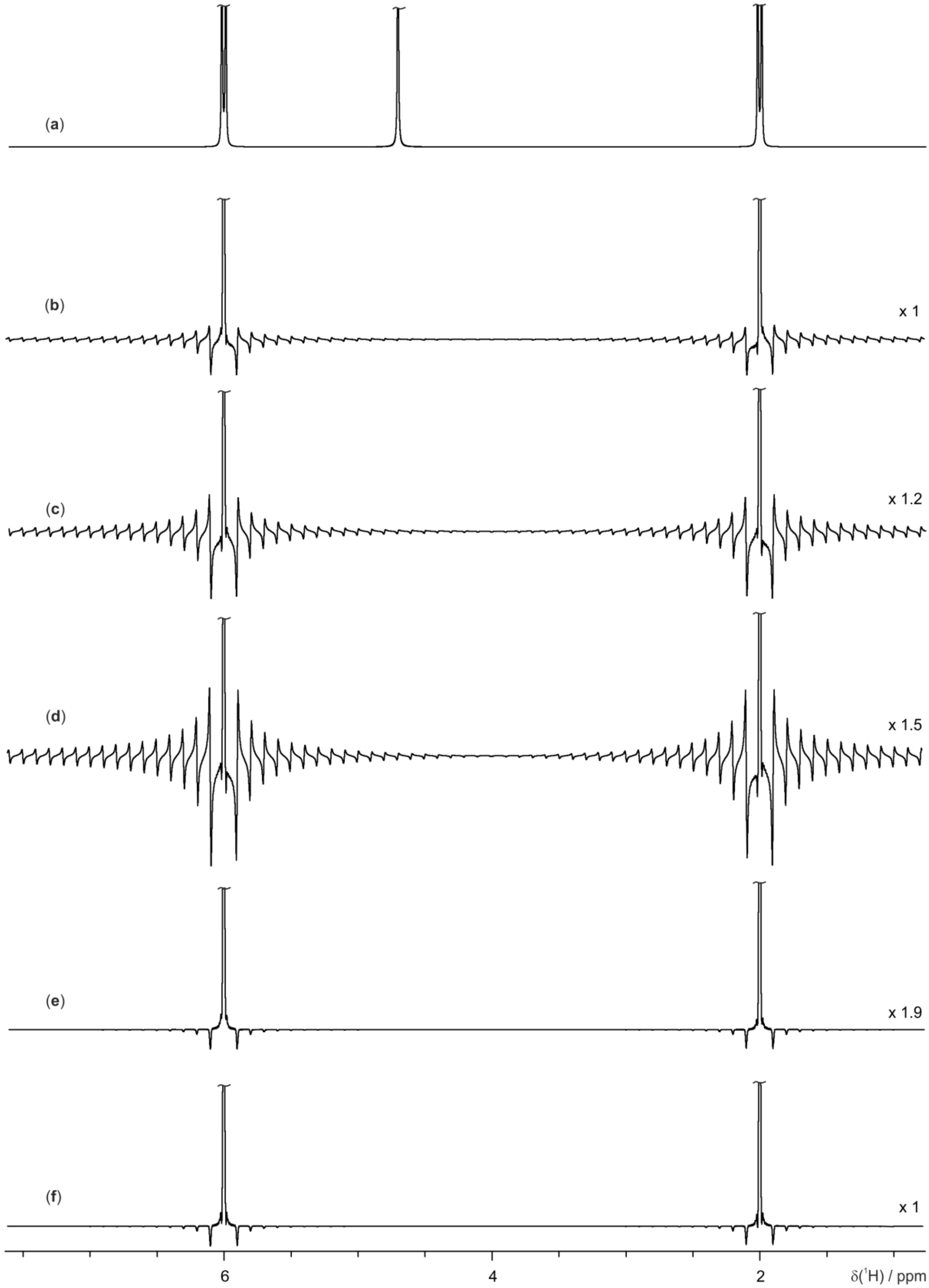

Figure S11: 500 MHz ^{1}H NMR spectra, simulated using Spinach software,[18] of an AX spin system with a signal at 4.7 ppm representing the solvent signal. (a) Conventional ^{1}H spectrum. (b) Concatenated WG_1-TSE-PSYCHE spectrum, (c) concatenated WG_2-TSE-PSYCHE spectrum, (d) concatenated WG_3-TSE-PSYCHE spectrum, (e) concatenated perfect-echo WG-TSE-PSYCHE spectrum, and (f) PSYCHE-iWG spectrum. The WG element used 3 ms rectangular selective pulses to target the solvent signal at 4.7 ppm. The PSYCHE element consisted of two consecutive saltire pulses of 10 kHz bandwidth and 30 ms duration, calibrated to achieve a 20° on-resonance flip angle. A simultaneous gradient of 1.5 G cm^{-1} was applied during the saltire pulses. For the concatenated WG_n-TSE-PSYCHE experiments, additional swept-frequency chirp 180° pulses of 10 ms, with a simultaneous spatial encoding gradient of 1 G cm^{-1}, surround the PSYCHE pulse. 10 chunks of 20 ms duration were acquired.

F. Pulse program for Bruker spectrometer

```
;Pure shift WATERGATE using iWG (selective 90 pulses) or iW5 (binomial W5) elements
;WATERGATE options include
;	ZGOPTION= -DW5
;	ZGOPTION= -DWG

;Wavemaker compatible (run 'wvm -a' before 'zg')

; 1D Pure shift experiment with interferogram acquisition
;	ZGOPTION= -DBS (Band-Selective)
;	ZGOPTION= -DZS (Zangger-Sterk)
;	ZGOPTION= -DdSALTIRE (PSYCHE)
;
;Data can be reconstructed using a ‘pshift’ macro available at http://nmr.chemistry.manchester.ac.uk
;Pulse sequence is also available for download from http://nmr.chemistry.manchester.ac.uk

; Developed by:
; NMR Methodology Group
; University of Manchester

;References:
;WATERGATE:
;		(1) M. Piotto, V. Saudek, V. Sklenar, J. Biomol. NMR, 2, 661-665, (1992)
;		(2) M. Liu, X. Mao, C. Ye, H. Huang, J. K. Nicholson, J.C. Lindon, J. Magn. Reson., 132, 125-129, (1998)
;Band-selection:
; (1) L. Castanar, P. Nolis, A. Virgili, T. Parella, Chem. Eur. J., 19, 17283-17286, (2013)
; (2) J. Ying, J. Roche, A. Bax, J. Magn. Reson., 241, 97-102, (2014)
; (3) R. W. Adams, L. Byrne, P. kiraly, M. Foroozabdeh, L. Paudel, M. Nilsson, J. Clayden, G. A. Morris, Chem. Commun., 50,
2512, (2014)
;Zangger-Sterk:
; (1) K. Zangger, H. Sterk, J. Magn. Reson., 124, 486-489, (1997)
;PSYCHE:
; (1) M. Foroozandeh, R. W. Adams, N. J. Meharry, D. Jeannerat, M. Nilsson, G. A. Morris, Angew. Chem. Int. Ed., 53, 6990 (2014)
;Interferogram:
; (1) K. Zangger, H. Sterk, J. Magn. Reson., 124, 486-489, (1997)
; (2) J. A. Aguilar, S. Faulkner, M. Nilsson, G. A. Morris, 49, 3901-3903, (2010)

;Avance NEO Version
;Topspin.4x
;
;$CLASS=HighRes
;$DIM=2D
;$TYPE=
;$SUBTYPE=
;$COMMENT=

#include <Avance.incl>
#include <Delay.incl>
#include <Grad.incl>

define delay tauA
define delay tauB

;DELAYS
"d0=0"
"in0=inf1/2"
"tauA=in0/2"
"tauB=dw*2*cnst4"
"d16=1m"
"d17=1m"

;PULSES
"p2=p1*2"
"p16=1m"
"p17=1m"
"p10=p40"
```

```
;CONSTANTS
"d11=30m"
#   ifdef WG
"d11=30m+1s/(1+cnst11)"
"d11=30m+1s/(1+cnst13)"
"d11=30m"
#endif

;WaveMaker
;sp11(p11):wvm:eg_rect1:f1 square90(cnst11 ms);
;sp13(p13):wvm:eg_rect2:f1 square90(cnst13 ms);

;SALTIRE PULSE
#   ifdef dSALTIRE
;Double Saltire pulse calculations
"cnst50=(cnst20/360)*sqrt((2*cnst21)/(p40/2000000))"
"p30=1000000.0/(cnst50*4)"
"cnst31= (p30/p1) * (p30/p1)"
"spw40=plw1/cnst31"
"spoff40=0"
#else
"d11=30m+1s/(1+cnst12)"
"d11=30m"
;sp12:wvm:eg_rsnob:f1 rsnob(cnst12 Hz; NPOINTS=10000)
"spoff12=bf1*(cnst1/1000000)-o1"
#   endif

;ACQUISITION
"acqt0=0"
baseopt_echo

1 ze
2 d11
  50u BLKGRAMP
  50u LOCKH_OFF
  d1 pl1:f1
  50u LOCKH_ON
  50u UNBLKGRAMP

3 p1 ph1

  tauB
  tauA
  50u

# ifdef WG
  p16:gp1
  d16 pl0:f1
  (p11:sp11 ph2:r):f1
  4u
  d12 pl1:f1
  (p2 ph5)
  4u
  d12 pl0:f1
  (p11:sp11 ph2:r):f1
  p16:gp1
  d16 pl1:f1
# endif

#ifdef W5
  p16:gp1
  d16 pl18:f1
  p27*0.087 ph4
  d19*2
  p27*0.206 ph4
  d19*2
  p27*0.413 ph4
  d19*2
  p27*0.778 ph4
  d19*2
```

```
  p27*1.491 ph4
  d19*2
  p27*1.491 ph5
  d19*2
  p27*0.778 ph5
  d19*2
  p27*0.413 ph5
  d19*2
  p27*0.206 ph5
  d19*2
  p27*0.087 ph5
  p16:gp1
  d16 pl1:f1
#endif

  50u
  tauA

  d0

  d17 pl0:f1
  p17:gp2
  d17
# ifdef BS
  10u
  (p12:sp12 ph3):f1
  10u
# endif
# ifdef ZS
  10u gron0
  (p12:sp12 ph3):f1
  10u groff
# endif
# ifdef dSALTIRE
  10u
 ( center (p40:sp40 ph3):f1 (p10:gp10) )
  10u
# endif
  d17
  p17:gp2
  d17

  50u

#ifdef W5
  p16:gp3
  d16 pl18:f1
  p27*0.087 ph6
  d19*2
  p27*0.206 ph6
  d19*2
  p27*0.413 ph6
  d19*2
  p27*0.778 ph6
  d19*2
  p27*1.491 ph6
  d19*2
  p27*1.491 ph7
  d19*2
  p27*0.778 ph7
  d19*2
  p27*0.413 ph7
  d19*2
  p27*0.206 ph7
  d19*2
  p27*0.087 ph7
  p16:gp3
  d16
#endif
```

```
# ifdef WG
  p16:gp3
  d16 pl0:f1
  (p13:sp13 ph4:r):f1
  4u
  d12 pl1:f1
  (p2 ph5)
  4u
  d12 pl0:f1
  (p13:sp13 ph4:r):f1
  p16:gp3
  d16
#endif

  50u pl1:f1

  d0
  50u BLKGRAMP

  go=2 ph31
  d11 mc #0 to 2 F1QF(id0)
exit

ph1= 0 0 0 0 1 1 1 1 2 2 2 2 3 3 3 3
ph2= 0
ph3= 0 1 2 3
ph4= 0
ph5= 2
ph6= 0
ph7= 2
ph31=0 2 0 2 3 1 3 1 2 0 2 0 1 3 1 3

;POWER LEVEL
;pl0 : zero power [0 W]
;pl1 : power level for pulse (default)
;sp12: f1 channel - power level for  selective 180 pulse
;pl18: f1 channel - power level for 3-9-19-pulse of the Watergate element
;sp40: f1 channel - power level for low-flip angle saltire pulse

;PULSE DURATION
;p1  : hard 90 pulse (high power)
;p12 : duration of selective 180 pulse (low power)
;p40 : duration of Saltire pulse (low power)

;GRADIENT DURATION
;p10 : duration of the weak gradient during saltire pulse [p10 = p40]
;p16 : duration of CTP gradients of hard 180 pulse       [1 ms]
;p17 : duration of CTP gradients of selective 180 pulse  [1 ms]

;DELAYS
;d0  : incremented delay
;d1  : relaxation delay; 1-5 * T1
;d16 : recovery delay for CTP gradients of hard 180 pulse      [1 ms]
;d17 : recovery delay for CTP gradients of selective 180 pulse  [1 ms]

;PULSE SHAPE
;spnam12: shape of the selective 180 pulse
;spnam40: file name for saltire pulse

;GRADIENT SHAPE
;gpnam1: SMSQ10.100
;gpnam2: SMSQ10.100
;gpnam3: SMSQ10.100
;gpnam10: RECT.1

;GRADIENT STRENGTH
;gpz0: Spatial Encoding gradient for ZS     [0.1-2%]
;gpz1: CTP gradient for first hard 180 pulse      [35%]
;gpz2: CTP gradient for selective 180 pulse [19%]
;gpz3: CTP gradient for second hard 180 pulse      [79%]
```

;gpz10: weak gradient during saltire pulse [1-3%]

;CONSTANTS
;cnst1: Chemical shift for the selective 180 Rsnob pulse [ppm]
;cnst4: number of drop points [4]
;cnst11: duration of first selective 90 pulses in WG[ms]
;cnst12: bandwidth of the selective 180 Rsnob pulse [Hz]
;cnst13: duration of second selective 90 pulses in WG [ms]
;cnst20: desired flip angle for saltire pulse element [degree, normally 10-50]
;cnst21: bandwidth of saltire pulse [Hz, normally 10000 Hz]

;OTHERS
;BS: set O1 on resonance on the frequency to be excited
;ZS: set O1 on in the center of the spectral window to be excited
;PSYCHE: set O1 on in the center of the spectral window to be excited
;td1 : number of t1 increments [16-64]
;in0 : 1/(2 * SW) = DW
;nd0 : 2
;MC2 : QF
;ns : 2 * n
;ds : 2

G. References

(1) Piotto, M.; Saudek, V.; Sklenář, V. Gradient-Tailored Excitation for Single-Quantum NMR Spectroscopy of Aqueous Solutions. *J. Biomol. NMR* **1992**, *2* (6), 661–665. https://doi.org/10.1007/BF02192855.

(2) Liu, M.; Mao, X. A.; Ye, C.; Huang, H.; Nicholson, J. K.; Lindon, J. C. Improved Watergate Pulse Sequences for Solvent Suppression in NMR Spectroscopy. *J. Magn. Reson.* **1998**, *132* (1), 125–129. https://doi.org/10.1006/jmre.1998.1405.

(3) Foroozandeh, M.; Adams, R. W.; Meharry, N. J.; Jeannerat, D.; Nilsson, M.; Morris, G. A. Ultrahigh-Resolution NMR Spectroscopy. *Angew. Chem. Int. Ed.* **2014**, *53* (27), 6990–6992. https://doi.org/10.1002/anie.201404111.

(4) Zangger, K.; Sterk, H. Homonuclear Broadband-Decoupled NMR Spectra. *J. Magn. Reson.* **1997**, *124* (2), 486–489. https://doi.org/10.1006/jmre.1996.1063.

(5) Ying, J.; Roche, J.; Bax, A. Homonuclear Decoupling for Enhancing Resolution and Sensitivity in NOE and RDC Measurements of Peptides and Proteins. *J. Magn. Reson.* **2014**, *241* (1), 97–102. https://doi.org/10.1016/j.jmr.2013.11.006.

(6) Castañar, L.; Nolis, P.; Virgili, A.; Parella, T. Full Sensitivity and Enhanced Resolution in Homodecoupled Band-Selective NMR Experiments. *Eur. J. Chem.* **2013**, *19* (51), 17283 – 17286. https://doi.org/10.1002/chem.201303235.

(7) Hwang, T. L.; Shaka, A. J. Water Suppression That Works. Excitation Sculpting Using Arbitrary Wave-Forms and Pulsed-Field Gradients. *J. Magn. Reson. - Ser. A* **1995**, *112* (2), 275–279. https://doi.org/10.1006/jmra.1995.1047.

(8) Garbow, J. R.; Weitekamp, D. P.; Pines, A. Bilinear Rotation Decoupling of Homonuclear Scalar Interactions. *Chem. Phys. Lett.* **1982**, *93* (5), 504–509. https://doi.org/10.1016/0009-2614(82)83229-6.

(9) Bertho, G.; Lordello, L.; Chen, X.; Lucas-Torres, C.; Oumezziane, I. E.; Caradeuc, C.; Baudin, M.; Nuan-Aliman, S.; Thieblemont, C.; Baud, V.; Giraud, N. Ultrahigh-Resolution NMR with Water Signal Suppression for a Deeper Understanding of the Action of Antimetabolic Drugs on Diffuse Large B-Cell Lymphoma. *J. Proteome Res.* **2022**, *21* (4), 1041–1051. https://doi.org/10.1021/acs.jproteome.1c00914.

(10) Kunjir, S.; Rodriguez-Zubiri, M.; Coeffard, V.; Felpin, F.-X.; Giraudeau, P.; Farjon, J. Merging Gradient-Based Methods to Improve Benchtop NMR Spectroscopy: A New Tool for Flow Reaction Optimization. *ChemPhysChem* **2020**, *21* (20), 2311–2319. https://doi.org/10.1002/cphc.202000573.

(11) Foroozandeh, M.; Adams, R. W.; Kiraly, P.; Nilsson, M.; Morris, G. A. Measuring Couplings in Crowded NMR Spectra: Pure Shift NMR with Multiplet Analysis. *Chem. Commun.* **2015**, *51* (84), 15410–15413. https://doi.org/10.1039/c5cc06293d.

(12) Ernst, R. R.; Anderson, W. A. Application of Fourier Transform Spectroscopy to Magnetic Resonance. *Rev. Sci. Instrum.* **1966**, *37*, 93–102. https://doi.org/10.1063/1.1719961.

(13) Adams, R. W. Pure Shift NMR Spectroscopy. *eMagRes* **2014**, *3* (4), 295–309. https://doi.org/10.1002/9780470034590.emrstm1362.

(14) Castañar, L.; Parella, T. Broadband 1H Homodecoupled NMR Experiments: Recent Developments, Methods and

Applications. *Magn. Reson. Chem.* **2015**, *53* (6), 399–426. https://doi.org/10.1002/mrc.4238.

(15) Zangger, K. Pure Shift NMR. *Prog. Nucl. Magn. Reson. Spectrosc.* **2015**, *86–87*, 1–20. https://doi.org/10.1016/j.pnmrs.2015.02.002.

(16) Castañar, L. Pure Shift 1H NMR: What Is Next? *Magn. Reson. Chem.* **2017**, *55* (1), 47–53. https://doi.org/10.1002/mrc.4545.

(17) Adams, R. W.; Holroyd, C. M.; Aguilar, J. A.; Nilsson, M.; Morris, G. A. “Perfecting” WATERGATE: Clean Proton NMR Spectra from Aqueous Solution. *Chem. Commun.* **2013**, *49* (4), 358–360. https://doi.org/10.1039/c2cc37579f.

(18) Hogben, H. J.; Krzystyniak, M.; Charnock, G. T. P.; Hore, P. J.; Kuprov, I. Spinach - A Software Library for Simulation of Spin Dynamics in Large Spin Systems. *J. Magn. Reson.* **2011**, *208* (2), 179–194. https://doi.org/10.1016/j.jmr.2010.11.008.
